# A safe and highly efficacious measles virus-based vaccine expressing SARS-CoV-2 stabilized prefusion spike

**DOI:** 10.1073/pnas.2026153118

**Published:** 2021-03-09

**Authors:** Mijia Lu, Piyush Dravid, Yuexiu Zhang, Sheetal Trivedi, Anzhong Li, Olivia Harder, Mahesh KC, Supranee Chaiwatpongsakorn, Ashley Zani, Adam Kenney, Cong Zeng, Chuanxi Cai, Chengjin Ye, Xueya Liang, Masako Shimamura, Shan-Lu Liu, Asuncion Mejias, Octavio Ramilo, Prosper N. Boyaka, Jianming Qiu, Luis Martinez-Sobrido, Jacob S. Yount, Mark E. Peeples, Amit Kapoor, Stefan Niewiesk, Jianrong Li

**Affiliations:** ^a^Department of Veterinary Biosciences, The Ohio State University, Columbus, OH 43210;; ^b^Center for Vaccines and Immunity, Abigail Wexner Research Institute at Nationwide Children’s Hospital, Columbus, OH 43205;; ^c^Department of Microbial Infection and Immunity, College of Medicine, The Ohio State University, Columbus, OH 43210;; ^d^Department of Surgery, College of Medicine, The Ohio State University, Columbus, OH 43210;; ^e^Texas Biomedical Research Institute, San Antonio, TX 78227;; ^f^Department of Pediatrics, College of Medicine, The Ohio State University, Columbus, OH 43210;; ^g^Infectious Disease Institute, The Ohio State University, Columbus, OH 43210;; ^h^Center for Retrovirus Research, The Ohio State University, Columbus, OH 43210;; ^i^Department of Microbiology, Molecular Genetics and Immunology, University of Kansas Medical Center, Kansas City, KS 66160

**Keywords:** SARS-CoV-2 vaccine, measles virus vector, prefusion spike

## Abstract

The current pandemic of COVID-19 caused by severe acute respiratory syndrome coronavirus 2 (SARS-CoV-2) highlights an urgent need to develop a safe, efficacious, and durable vaccine. Using a measles virus (rMeV) vaccine strain as the backbone, we developed a series of recombinant attenuated vaccine candidates expressing various forms of the SARS-CoV-2 spike (S) protein and its receptor binding domain (RBD) and evaluated their efficacy in cotton rat, IFNAR^−/−^mice, IFNAR^−/−^-hCD46 mice, and golden Syrian hamsters. We found that rMeV expressing stabilized prefusion S protein (rMeV-preS) was more potent in inducing SARS-CoV-2–specific neutralizing antibodies than rMeV expressing full-length S protein (rMeV-S), while the rMeVs expressing different lengths of RBD (rMeV-RBD) were the least potent. Animals immunized with rMeV-preS produced higher levels of neutralizing antibody than found in convalescent sera from COVID-19 patients and a strong Th1-biased T cell response. The rMeV-preS also provided complete protection of hamsters from challenge with SARS-CoV-2, preventing replication in lungs and nasal turbinates, body weight loss, cytokine storm, and lung pathology. These data demonstrate that rMeV-preS is a safe and highly efficacious vaccine candidate, supporting its further development as a SARS-CoV-2 vaccine.

## Significance

Measles virus (MeV) vaccine is one of the safest and most efficient vaccines with a track record in children. Here, we generated a panel of rMeV-based vaccines with severe acute respiratory syndrome coronavirus 2 (SARS-CoV-2) S antigens inserted near 3′ of the MeV genome. The rMeV expressing a soluble stabilized, prefusion spike (preS) is much more potent in triggering SARS-CoV-2–specific neutralizing antibody than rMeV-based full-length S vaccine candidate. A single dose of rMeV-preS is sufficient to induce high levels of SARS-CoV-2 antibody in animals. Furthermore, rMeV-preS induces high levels of Th1-biased immunity. Hamsters immunized with rMeV-preS were completely protected against SARS-CoV-2 challenge. Our results demonstrate rMeV-preS is a safe and highly efficacious bivalent vaccine candidate for SARS-CoV-2 and MeV.

In December 2019, a novel coronavirus disease (COVID-19) was first identified in Wuhan City, Hubei Province, People’s Republic of China. The causative agent was named severe acute respiratory syndrome coronavirus 2 (SARS-CoV-2). On 11 March 2020 the World Health Organization (WHO) declared COVID-19 a global pandemic (1–3). It spread rapidly within China and swept into at least 200 countries within 3 mo. Symptoms are primarily pneumonia, as with two other important human coronaviruses (CoVs), SARS-CoV-1 and Middle East respiratory syndrome (MERS)-CoV (1–3). As of 1 February 2021, more than 102,399,513 cases had been reported worldwide, with 2,217,005 deaths (∼2.2% mortality). There is an urgent need to develop a safe and efficacious vaccine to protect the populace from this new virus. Globally, more than 300 SARS-CoV-2 vaccine candidates are in preclinical development (4–6) and at least 30 vaccine candidates have entered human clinical trials (4, 5, 7, 8). Among them, vaccines based on messenger RNA (mRNA), inactivated virus, and adenovirus vectors (Ad5-nCoV and ChAdOx1) are now in phase III clinical trials. Excitingly, preliminary results indicate that these vaccines are highly efficacious, reaching 90 to 95% effectiveness against SARS-CoV-2 infection in some cases. The durability of the protection conferred by these vaccine candidates is unknown. Although these vaccine candidates are highly promising, exploration of other vaccine platforms is needed.

The CoV spike (S) protein is the main target for neutralizing antibodies that inhibit infection and prevent disease. As such, the S protein is the primary focus for CoV vaccine development (9, 10). The CoV S protein is a class I fusion protein trimer that is incorporated into virions as they bud into the endoplasmic reticulum–Golgi intermediate compartment. For SARS-CoV-2, S is cleaved into S1 and S2 subunits by furin before the virion is released. The S1 subunit contains the receptor-binding domain (RBD) that attaches to the hACE2 receptor on the surface of a target cell. The S2 subunit is further cleaved by TMPRSS2 (or cathepsin L/B) and possesses the membrane-fusing activity (9, 11, 12). Both S and its RBD have been shown to be immunogenic for many CoVs (13–15). The native S in the virion is in its “prefusion” form. Upon triggering, the prefusion S (preS) undergoes significant conformational changes to insert its fusion peptide into the target cell membrane and bring the virion and cell membranes together, arriving at its postfusion S form as it causes the membranes to fuse. For paramyxoviruses, pneumoviruses, and HIV, it has been shown that prefusion forms of glycoprotein are more potent in inducing neutralizing antibodies than their postfusion forms (16–20). Currently, whether the SARS-CoV-2 preS protein is more immunogenic than the postfusion S protein is unknown.

Live attenuated measles virus (MeV) vaccine has been one of the safest and most efficient human vaccines and has been used in children since the 1960s (21, 22). Worldwide MeV vaccination campaigns have been very successful in controlling measles. MeV is an enveloped nonsegmented negative-sense RNA virus that belongs to the genus *Morbillivirus* within the Paramyxoviridae family. MeV is an excellent vector to deliver vaccines for human pathogens primarily because of its high safety, efficacy, and long-lived immunity (22, 23). MeV has previously been shown to be a highly efficacious vaccine vector for many viral diseases such as HIV (24, 25), SARS-CoV-1 (26, 27), MERS-CoV (28, 29), respiratory syncytial virus (30), hepatitis B and C viruses (31), influenza virus (30, 32), chikungunya virus (CHIKV) (33), and flaviviruses (Zika virus, dengue virus, West Nile virus, and yellow fever virus) (34–36). Recent human clinical trials have demonstrated that an recombinant MeV (rMeV)-based CHIKV vaccine is safe and highly immunogenic in healthy adults, even in the presence of preexisting anti-MeV vector immunity (33).

In this study, we developed a series of rMeV-based vaccine candidates expressing different forms of the SARS-CoV-2 S protein and evaluated them in cotton rats, IFNAR^−/−^mice, IFNAR^−/−^-hCD46 mice, and golden Syrian hamsters. We found that all SARS-CoV-2 S antigens are highly expressed by the MeV vector. Among these vaccine candidates, rMeV expressing stabilized preS (rMeV-preS) and full-length S (rMeV-S) proteins were the most potent in triggering SARS-CoV-2–specific antibodies. Animals immunized with rMeV-preS induced the highest level of neutralizing antibodies that were higher than convalescent sera of patients recovered from COVID-19, and the highest Th1-biased T cell immune response. Furthermore, hamsters immunized with rMeV-preS provided complete protection against SARS-CoV-2 challenge and lung pathology.

## Results

### Recovery of Recombinant MeV Expressing SARS-CoV-2 S Antigens.

**Fig. 1.**
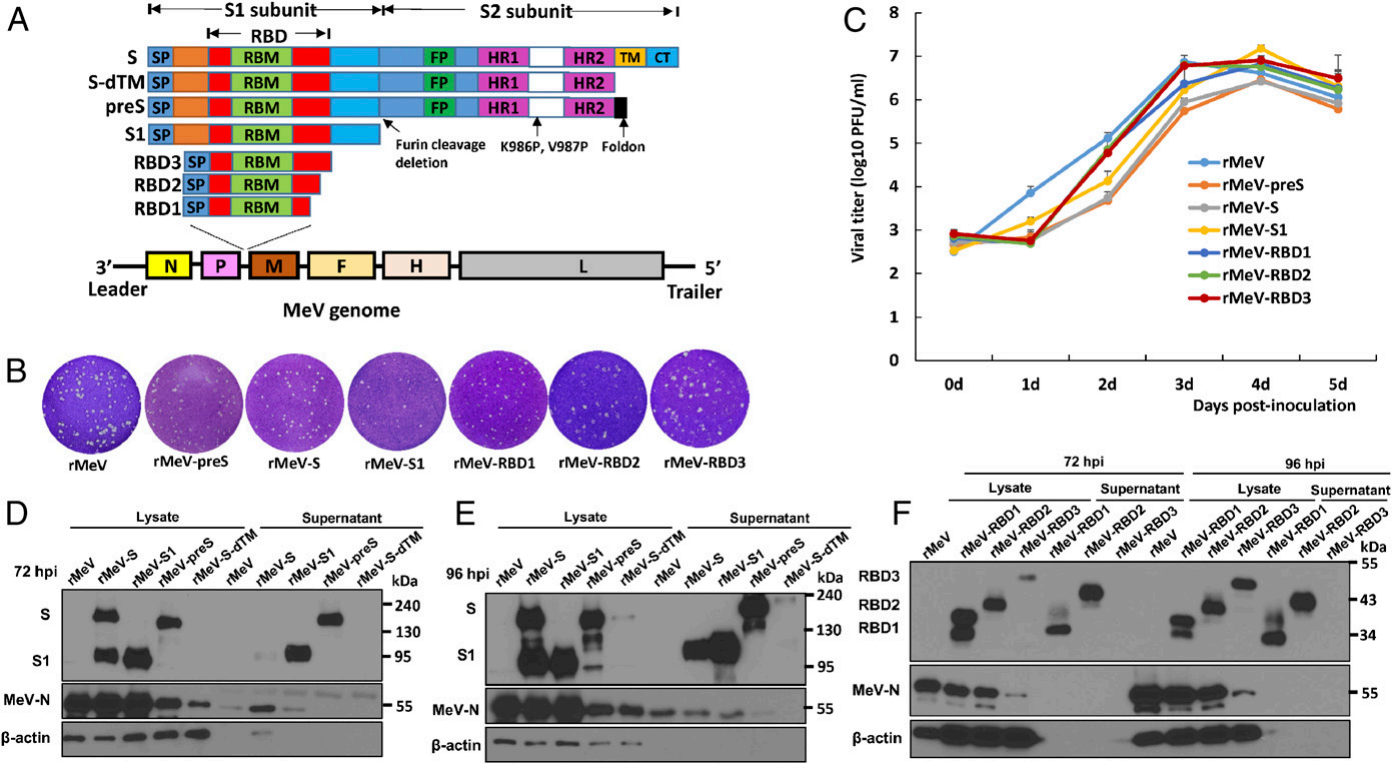
Recovery and characterization of rMeV expressing SARS-CoV-2 S proteins. (*A*) Strategy for insertion of SARS-CoV-2 S and its variants to MeV genome. The codon optimized full-length S, preS, S-dTM, S1, RBD1, RBD2, and RBD3 were amplified by PCR and inserted into the same position at the gene junction between P and M in the genome of the MeV Edmonston vaccine strain. The domain structure of S protein is shown: SP, signal peptide; RBD, receptor-binding domain; RBM, receptor-binding motif; FP, fusion peptide; HR, heptad repeat; CH, central helix; TM, transmembrane domain; CT, cytoplasmic tail. The organization of negative-sense MeV genome is shown. Le, leader sequence; N, nucleocapsid gene; P, phosphoprotein gene; M, matrix protein gene; F, fusion protein gene; H, hemagglutinin protein gene; L, large polymerase gene; Tr, trailer sequence. (*B*) The plaque morphology of rMeV expressing SARS-CoV-2 S antigens. All plaques were developed after 5 d of incubation in Vero CCL-81 cells. (*C*) Multistep growth curve. Confluent Vero CCL81 cells in 12-well plates were infected with each virus at an MOI of 0.01. After 1 h of absorption, fresh DMEM with 2% fetal bovine serum was added. The cell culture supernatants and cell lysates were harvested and combined, and virus titers were determined by plaque assay. Data are geometric mean titers (GMT) ± SD from *n* = 3 biologically independent experiments. (*D* and *E*) Analysis of SARS-CoV-2 S and S1 protein expression in cell lysate and supernatants by Western blot. Vero CCL81 cells in 12-well plates were infected with each recombinant virus at an MOI of 0.01. At 72 h (*D*) or 96 h (*E*) postinfection, cells were lysed in 300 μL of lysis buffer, and 10 μL of lysate or supernatant was analyzed by sodium dodecyl sulfate polyacrylamide gel electrophoresis and blotted with anti–SARS-CoV-2 S protein antibody (*Top*), MeV N antibody (*Middle*), or β-actin antibody (*Bottom*). (*F*) Analysis of RBD protein expression by Western blot. Ten microliters of lysate or supernatant at 72 and 96 h postinfection was analyzed. Western blots shown are the representatives of three independent experiments.

We have developed a yeast-based recombination system for rapidly constructing complementary DNA (cDNA) clones of rMeV expressing foreign genes such as the SARS-CoV-2 S antigens. Six overlapping DNA fragments (designated a to f) spanning the full-length MeV Edmonston vaccine strain and a SARS-CoV-2 gene annealing to the junction between the P and M genes were ligated into the pYES2 vector in a single step mediated by DNA recombinases present in yeast (*SI Appendix*, Fig. S1). Using this strategy, we constructed a series of rMeV vaccine vectors expressing eight variants of the SARS-CoV-2 S protein: 1) full-length S (S), 2) deletion of the transmembrane domain and cytoplasmic tail reflecting the soluble ectodomain (S-dTM), 3) S1 subunit (S1), 4) three different lengths of RBD (RBD1, RBD2, and RBD3) of S, and 5) a prefusion-stabilized soluble ectodomain with deletion of the furin cleavage site, two proline mutations (amino acids 986 and 987), and a self-trimerizing T4 fibritin trimerization motif replacing its transmembrane and cytoplasmic domains (preS) (9) (Fig. 1*A*). All rMeV viruses were recovered from full-length genome cDNAs using the standard reverse genetics system and plaque-purified. To confirm that the recombinant viruses indeed contained the target gene, viral genomic RNA was extracted followed by RT-PCR using primers annealing to the flanking MeV P and M genes. PCR products were sequenced, confirming that S and its variants were inserted into the MeV genome between the P and M genes. Finally, the entire genome of each recombinant virus was sequenced to confirm that no additional mutations had been introduced. Compared to the parental rMeV, all recombinant viruses formed relatively smaller plaques (Fig. 1*B*) and exhibited delayed syncytia formation and cytopathic effects (CPE) (*SI Appendix*, Fig. S2). A multistep replication curve showed that these recombinant viruses had delayed replication kinetics in Vero CCL81 cells (Fig. 1*C*). However, the peak titer of rMeV-S1 (10^7.2^ plaque-forming units [PFU]/mL) was higher than that of the parental rMeV (10^6.9^ PFU/mL). Three recombinant viruses (rMeV-RBD1, RBD2, and RBD3) grew to titers comparable to the parental rMeV in Vero CCL-81 cells, whereas rMeV-S and rMeV-preS had 0.3 to 0.5 log reductions in peak titer. These results suggest that insertion of near-full-length SARS-CoV-2 S genes into the MeV genome further attenuates MeV replication.

### SARS-CoV-2 S Proteins Are Highly Expressed by the rMeV Vector.

We examined the expression of the SARS-CoV-2 S proteins by rMeV in confluent Vero CCL81 cells inoculated at a multiplicity of infection (MOI) of 0.01. Cell culture supernatants and lysates were harvested at 72 and 96 h postinfection and analyzed by Western blot using antibody against SARS-CoV-2 S1 protein or MeV N protein. As expected, two proteins with molecular weights of 190 and 95 kDa were detected in rMeV-S–infected cells at 72 h, reflecting the full-length S and cleaved S1 (Fig. 1*D*). In rMeV-preS–infected cells, the 180-kDa uncleaved, stabilized preS protein was detected, somewhat smaller because it lacks the transmembrane and cytoplasmic domains. In rMeV-S1–infected cells, the 95-kDa S1 protein was detected. The preS and S1 but not the full-length S were also secreted into the culture medium.

**Fig. 2.**
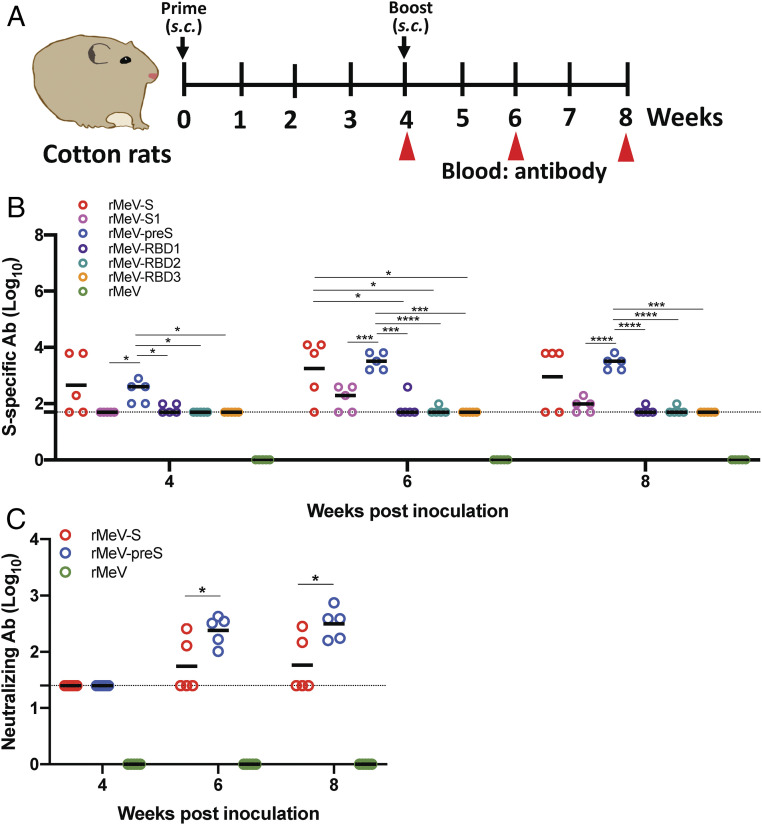
Immunogenicity of rMeVs expressing SARS-CoV-2 antigens in cotton rats. (*A*) Immunization schedule in cotton rats. Cotton rats (*n* = 5) were inoculated subcutaneously with phosphate-buffered saline or 4 × 10^5^ PFU of each of the rMeV-based vaccine candidate. Four weeks later, cotton rats were boosted with 10^6^ PFU of each virus. Serum samples were collected at weeks 4, 6, and 8 for antibody detection. (*B*) Measurement of SARS-CoV-2 S-specific antibody by ELISA. Highly purified preS protein was used as the coating antigen for the ELISA. Dotted line indicates the detectable level at the lowest dilution. (*C*) Measurement of SARS-CoV-2–specific neutralizing antibody. Antibody titer was determined by a plaque reduction neutralization assay. Dotted line indicates the detectable level at the lowest dilution. Data are expressed as the geometric mean titers (GMT) of five cotton rats ± SD. Data were analyzed using two-way ANOVA (*P < 0.05; ***P < 0.001; ****P < 0.0001).

By 96 h postinfection protein expression had increased (Fig. 1*D*). Although the S1 subunit from rMeV-S–infected cells had not been detected in the supernatant at 72 h, it was at 96 h (Fig. 2*E*). At both times, much of the S protein had been cleaved to its active form but the release of S1 into the supernatant at the later time suggests that some of the metastable cleaved S protein had either triggered spontaneously or following engagement with its receptor on a neighboring cell. Triggering releases S1 and allows S2 to refold, engaging with the target cell membrane and causing fusion between the two membranes.

RBD1 (34 kDa), RBD2 (40 kDa), and RBD3 (45 kDa) proteins were produced by their respective rMeV vector-infected cells (Fig. 2*F*), consistent with their predicted molecular weights. High levels of RBD1 and RBD2 were secreted into the cell culture supernatant. These results demonstrated that all of these SARS-CoV-2 S antigens were highly expressed by the rMeV vector, with the exception of S-dTM (Fig. 2*E*), which was not pursued further. The extensive fusion CPE observed at both 72 and 96 h (*SI Appendix*, Fig. S2) is most likely due to MeV, which causes this type of CPE. Interestingly, the CPE did not impair the production of most versions of the S protein over the 96 h of the experiment.

### rMeV-Expressed S and preS Are Highly Immunogenic in Cotton Rats.

Cotton rats (*Sigmodon hispidus*) are a susceptible model for MeV infection (37). Thus, we first tested the immunogenicity of these rMeV-based SARS-CoV-2 vaccine candidates in cotton rats (Fig. 2*A*). Four-week-old specific-pathogen-free cotton rats were immunized subcutaneously with 4 × 10^5^ PFU of each rMeV-based SARS-CoV-2 vaccine candidate and boosted with 2 × 10^6^ PFU of the same vaccine candidate 4 wk later. Sera were collected at weeks 4, 6, and 8, and S-specific antibodies were detected by enzyme-linked immunosorbent assay (ELISA) using preS protein as the antigen. By week 4, all five cotton rats in the rMeV-preS group had developed S-specific antibodies, whereas only three out of five cotton rats in the rMeV-S group had (Fig. 2*B*). However, antibodies were detectable at the lowest dilution in most cotton rats in the rMeV-S1 and RBD1-3 groups. After the booster immunization, antibodies in the rMeV-preS group were uniformly high, whereas three cotton rats in rMeV-S had high antibody titers and two had low antibody titers. Despite the booster immunization, antibody titers in the rMeV-S1 group remained low and antibody titers in rMeV-RBD1-3 were at the minimum detectable level (Fig. 2*B*).

The functional activities of the antibodies in sera from the two groups with the most antibody to S, rMeV-S and rMeV-preS, were tested for their ability to neutralize live SARS-CoV-2, in comparison to the rMeV group. Neutralizing antibody titers in the rMeV-preS group were significantly higher than those in the rMeV-S group (*P* < 0.05), on average 5.5-fold higher (Fig. 2*C*). Therefore, in the MeV expression system, preS is the most effective immunogen for inducing neutralizing antibodies in the cotton rat.

### rMeV-preS Is Highly Immunogenic in IFNAR1^−/−^-hCD46 Mice and Induces High Levels of Th1-Biased T Cell Immune Responses.

**Fig. 3.**
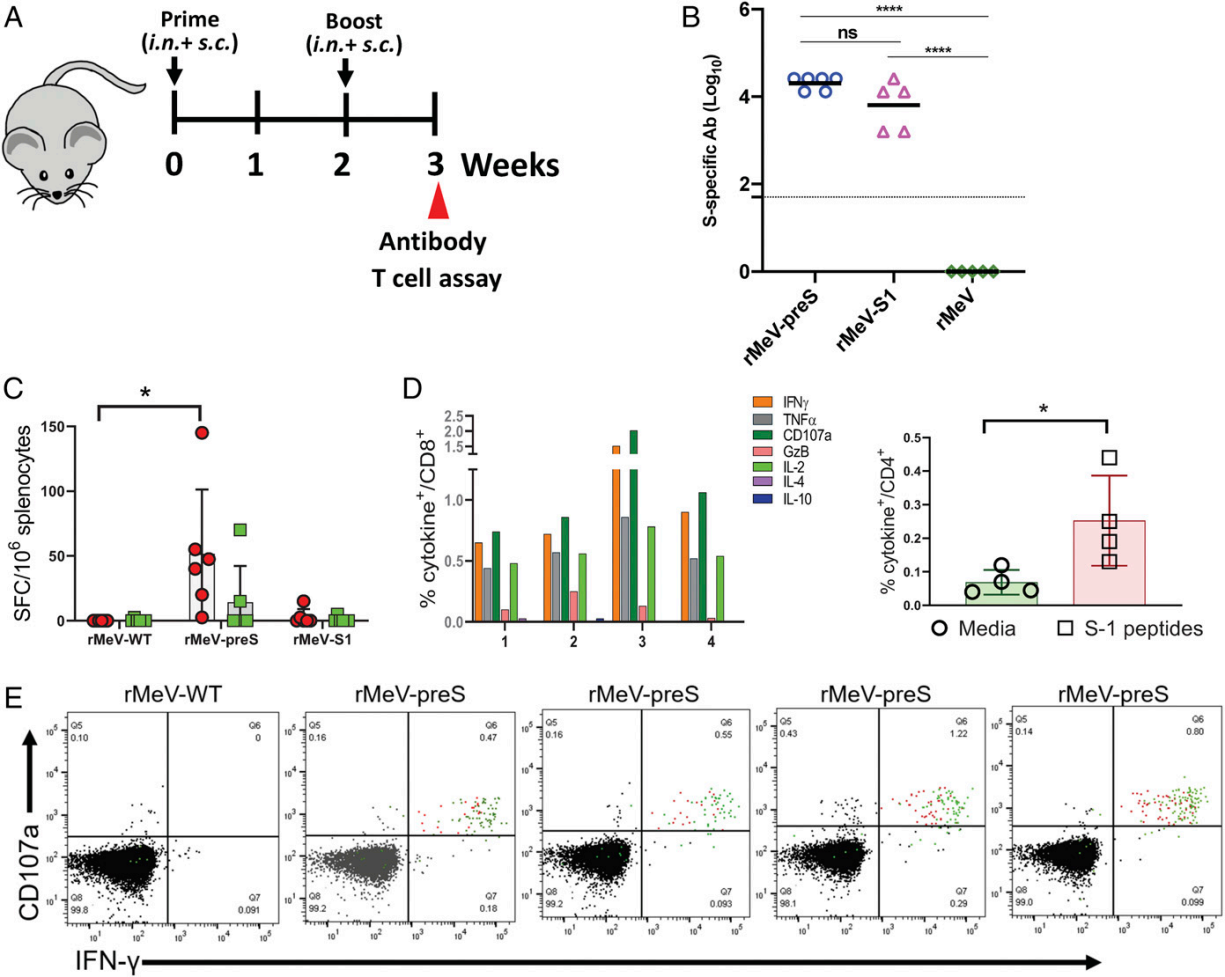
rMeV-preS is highly immunogenic in IFNAR^−/−^-hCD46 mice and induces strong Th1-biased T cell immune responses. (*A*) Immunization schedule. IFNAR^−/−^-hCD46 mice (*n* = 5 or 6) were inoculated with 8 × 10^5^ PFU of rMeV, rMeV-preS, or rMeV-S1. Two weeks later, mice were boosted with 6 × 10^5^ PFU of each virus. Half the dose was delivered subcutaneously and the other half was delivered intranasally. Serum samples were collected at week 3 for antibody detection. Mice were killed at week 3 for the T cell assays. (*B*) Measurement of SARS-CoV-2 S-specific antibody by ELISA. Highly purified preS protein was used as the coating antigen for ELISA. Dotted line indicates the detectable level at the lowest dilution. Data were analyzed using one-way ANOVA (****P < 0.0001; ns indicates no significant difference; *P* > 0.05). (*C*) ELISpot quantification of IFN-γ–producing T cells. Spot forming cells (SFC) were quantified after the cells were stimulated by peptides representing N (S1 peptides, red) and C (S2 peptides, green) termini of SARS-CoV-2 spike protein. Data are means of five mice ± SD. *P < 0.05 as determined by unpaired *t* test. (*D*) Cytokine expression in CD8^+^ and CD4^+^ splenocytes. Splenocytes of four rMeV-preS–vaccinated mice with highest SFC were stimulated ex vivo for 5 h with pools of S1 peptides representing the N-terminal of SARS-CoV-2 S protein (5 μg/mL each) in an intracellular cytokine staining assay. Frequencies of CD4^+^ T cells expressing cytokines represent CD4^+^ T cells expressing IFN-γ, TNF-α, or IL-2. *P < 0.05 as determined by Student's *t* test. (*E*) Flow plots of cytokine production. Antigen-stimulated CD8^+^ T cells in one rMeV vector-immunized and four rMeV-preS–immunized mice. CD8^+^ T cells expressing CD107a and IFN-γ are shown as red dots and cells also expressing TNF-α are shown as green dots.

MeV vaccine strains can use several receptors (human CD46, CD150, and Nectin 4) to infect different cell types (22). Type-I interferon receptor subunit 1 (IFNAR1) knockout, human CD46 transgenic mice (IFNAR1^−/−^-hCD46) mice can be robustly infected by MeV and have been used as a model to test the efficacy of many rMeV-based vaccine candidates (38). Thus, rMeV-preS and rMeV-S1 were tested in IFNAR1^−/−^-hCD46 mice to determine if they are immunogenic (Fig. 3*A*). Six-week-old IFNAR1^−/−^-hCD46 mice were immunized with 8 × 10^5^ PFU of each vaccine candidate (half subcutaneous and half intranasal) and at week 2 were boosted with the same vaccine candidate at a dose of 6 × 10^5^ PFU. Sera were collected at week 3 and antibody to preS was quantified by ELISA. We observed that rMeV-S1 induced higher antibody in IFNAR1^−/−^-hCD46 mice than in cotton rats. However, rMeV-preS induced more antibody than rMeV-S1, but the difference was not significant (*P* > 0.05) (Fig. 3*B*). These results suggest that rMeV-S1 may replicate more robustly in the presence of hCD46 receptor than it did in the cotton rat, where it induced a lower level of antibody.

At week 3, all groups were euthanized and their splenocytes were used to characterize vaccine-induced T cell immunity. We first quantified SARS-CoV-2 antigen-specific interferon gamma (IFN-γ)-producing T cells by enzyme-linked immune absorbent spot (ELISpot). Mice immunized with rMeV-preS had significantly higher frequencies of S1 peptide-specific IFN-γ–producing T cells compared to the control mice vaccinated with rMeV vector (*P* < 0.05) (Fig. 3*C*). Upon stimulation with peptide pools spanning the S1 subunit, five out of six mice in the rMeV-preS group showed a strong antigen-specific IFN-γ–producing T cell response, whereas only two out of six mice in the rMeV-S1 group showed a weak T cell response (Fig. 3*C*). When S2 peptide pools were used for stimulation, only two of six mice in the rMeV-preS group had a strong IFN-γ–producing T cell response (Fig. 3*C*), indicating that the vaccine candidate induced T cells primarily targeting the N terminus of the SARS-CoV-2 S protein. To further characterize the nature of vaccine-induced T cells, four mice with the strongest IFN-γ–producing T cell responses in the rMeV-preS group were analyzed using flow cytometry and intracellular cytokine staining (Fig. 3 *D* and *E*). Th1 cells, which produce cytokines such as IFN-γ, tumor necrosis factor alpha (TNF-α), and interleukin (IL)-2, play an important role in protection against viral infection (39). After peptide stimulation ex vivo, CD8^+^ T cells producing one or more of the three signature Th1 cytokines, IFN-γ, TNF-α, and IL-2, were detected in all four mice immunized with rMeV-preS (Fig. 3 *D* and *E*). Moreover, antigen-specific cytokine-producing CD4^+^ T cells were also detected but at lower frequencies representing 0.1 to 0.5% of the total CD4^+^ T cells (Fig. 3*D*). Together, these data suggest that rMeV-preS vaccine candidate is capable of inducing robust T cell immunity that is predominated by CD8^+^ T cells capable of producing Th1 cytokines.

### A Single Immunization of rMeV-preS Induces a High Level of Antibody in IFNAR1^−/−^ Mice.

Recently, it was shown that type-I interferon, but not the hCD46, is the barrier for MeV infection in mice (38). IFNAR1^−/−^ mice can be readily infected by MeV (38). Thus, we compared the effectiveness of single immunization and booster immunization in inducing S-specific antibody in IFNAR1^−/−^ mice. For the single immunization group, IFNAR1^−/−^ mice were immunized with 8 × 10^5^ PFU of rMeV-preS (half subcutaneous and half intranasal). For the booster immunization group, IFNAR1^−/−^ mice were immunized with 8 × 10^5^ PFU of rMeV-preS and were boosted at the same dose 4 wk later (*SI Appendix*, Fig. S3*A*). At week 7, S-specific antibody in the booster immunization group was significantly higher than the single immunization group (*P* < 0.01) (*SI Appendix*, Fig. S3*B*). At week 8, there was no significant difference between these two groups (*P* > 0.05) (*SI Appendix*, Fig. S3*B*). This result suggests that a single immunization of rMeV-preS may be sufficient to induce a high level of SARS-CoV-2–specific antibody.

### rMeV-preS Is Highly Immunogenic in Golden Syrian Hamsters.

**Fig. 4.**
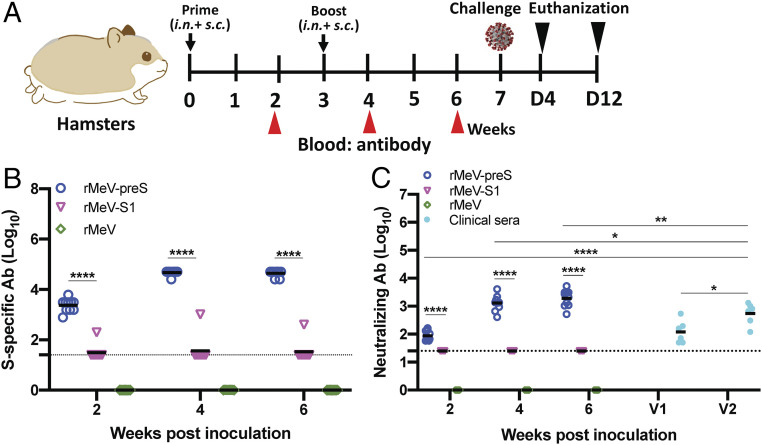
rMeV-preS is highly immunogenic in golden Syrian hamsters. (*A*) Immunization schedule in hamsters. Four-week-old female golden Syrian hamsters (*n* = 10) were immunized with 8 × 10^5^ PFU (half subcutaneous and half intranasal) of rMeV-preS, rMeV-S1, parental rMeV, or phosphate-buffered saline. Hamsters were boosted 3 wk later. At weeks 2, 4, and 6, sera were collected for antibody detection. At week 7, hamsters were challenged with 10^5^ PFU of SARS-CoV-2. Unimmunized, unchallenged controls were inoculated with DMEM. (*B*) Measurement of SARS-CoV-2 S-specific antibody. Highly purified preS protein was used as coating antigen for ELISA. Dotted line indicates the detectable level at the lowest dilution. (*C*) Measurement of SARS-CoV-2–specific neutralizing antibody. Antibody titer was determined by a plaque reduction neutralization assay. Human convalescent sera from acute infection (V1) and recovered COVID-19 patients (V2) were used as side-by-side controls. Data are expressed as the geometric mean titers (GMT) of 10 hamsters. Dotted line indicates the detectable level at the lowest dilution. Data were analyzed using two-way ANOVA (*P < 0.05; **P < 0.01; ****P < 0.0001).

Golden Syrian hamsters are an excellent animal model to evaluate SARS-CoV-2 pathogenesis and the efficacy of vaccine candidates or antiviral drugs. Early studies also suggest that golden Syrian hamsters are susceptible to MeV infection (40, 41). However, the optimal route for MeV immunization in hamsters is unknown. Thus, we chose the combination of intranasal and subcutaneous route for immunization in order to achieve maximal levels of immune responses. We chose rMeV-S1 to compare with rMeV-preS in the hamster study as rMeV-S1 induced good antibody responses in IFNAR1^−/−^-hCD46 mice (Fig. 3*B*) and grew to the highest titer in Vero cells (Fig. 1*C*). Ten 4-wk-old golden Syrian hamsters in each group were first immunized with 8 × 10^5^ PFU of the parental rMeV, rMeV-preS, or rMeV-S1 and boosted with the same dose 3 wk later (Fig. 4*A*). High antibody titers were detected in all 10 hamsters in the rMeV-preS group at week 2 after a single-dose vaccination. After a booster immunization at week 3, antibody titers further increased at weeks 4 and 6 (Fig. 4*B*). However, only 1 out of 10 hamsters in the rMeV-S1 group produced a robust antibody response (Fig. 4*B*). As expected, neutralizing antibodies in the rMeV-preS group were detectable at week 2 and increased at weeks 4 and 6, whereas neutralizing antibodies in the rMeV-S1 group remained at minimally detectable levels (Fig. 4*C*).

We compared the level of neutralizing antibody induced by rMeV-preS in hamsters with that induced in acute and convalescent sera collected from six COVID-19 patients at two time points: once diagnosis of SARS-CoV-2 was confirmed (V1) and 30 d later (V2). As expected, antibody titer of convalescent sera from the recovered COVID-19 patients was significantly higher than the titer of sera collected from the same patients during acute infection (*P* < 0.05) (Fig. 4*C*). Importantly, neutralizing antibody titers at weeks 4 and 6 in rMeV-preS–immunized hamsters were significantly higher than these random human convalescent sera (*P* < 0.05, *P* < 0.01) (Fig. 4*C*). These results confirm that rMeV-preS is highly immunogenic.

### rMeV-preS Vaccination Provides Complete Protection Against SARS-CoV-2 Replication in Golden Syrian Hamsters.

**Fig. 5.**
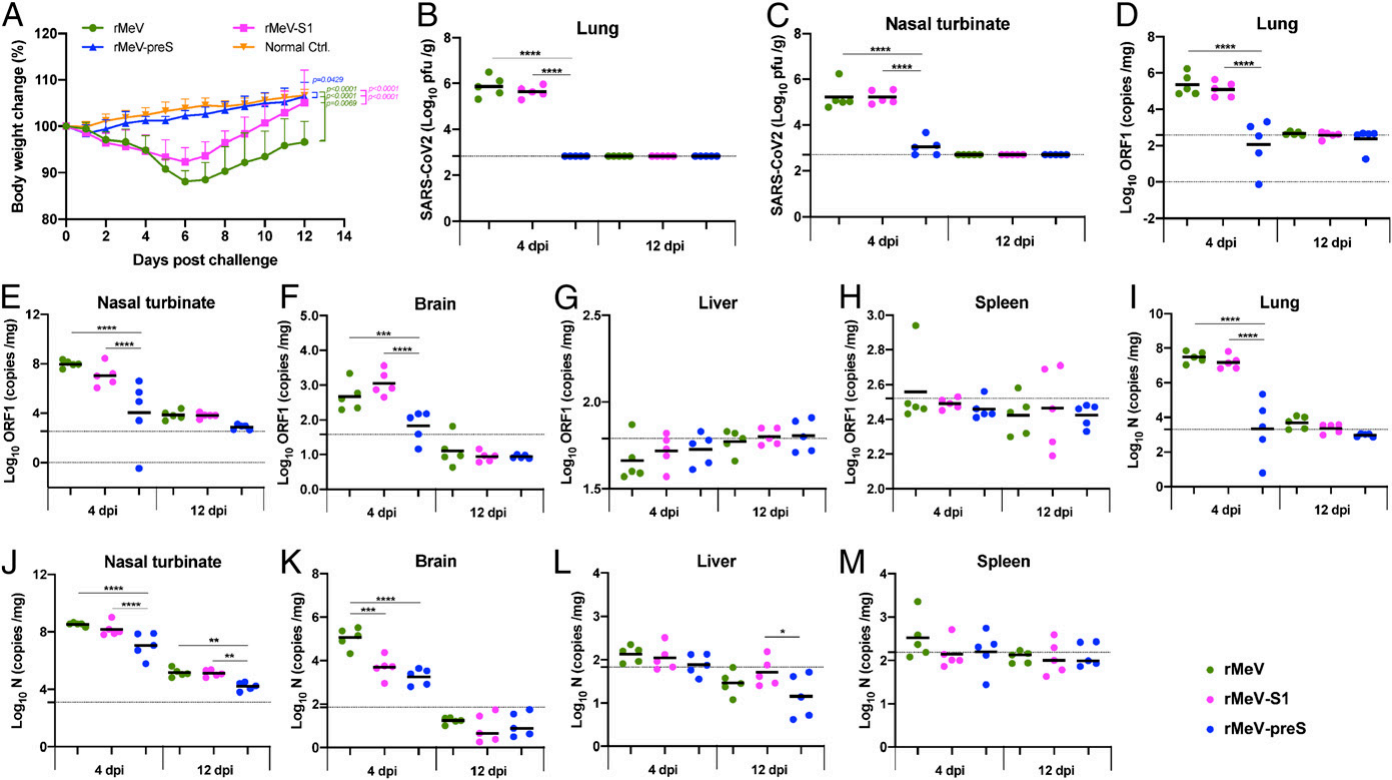
rMeV-preS provides complete protection against SARS-CoV-2 challenge in golden Syrian hamsters. (*A*) Dynamics of hamster body weight changes after SARS-CoV-2 challenge. The body weight for each hamster was measured daily and expressed as percentage of body weight at the challenge day. From days 0 to 4, the average body weight of 10 hamsters (*n* = 10) in each group is shown. From days 5 to 12, the average body weight of five hamsters (*n* = 5) in each group is shown. SARS-CoV-2 titer in lungs (*B*) and nasal turbinate (*C*). At day 4 after challenge, five hamsters from each group were killed and lungs and nasal turbinates were collected for virus titration by plaque assay. At day 12, the remaining five hamsters in each group were killed. Viral titers are the geometric mean titer (GMT) of five animals ± SD. The limit of detection (LoD) is 2.7∼2.8 Log_10_ PFU per gram of tissue (dotted line). SARS-CoV-2 genome RNA copies in lungs (*D*), nasal turbinate (*E*), brain (*F*), liver (*G*), and spleen (*H*). Total RNA was extracted from the homogenized tissue using TRIzol reagent. SARS-CoV-2 genome copies were quantified by real-time RT-PCR using primers annealing to the 5′ end of the genome. SARS-CoV-2 subgenomic RNA copies in lungs (*I*), nasal turbinate (*J*), brain (*K*), liver (*L*), and spleen (*M*). SARS-CoV-2 subgenomic RNA copies were quantified by real-time RT-PCR using primers annealing to the N gene at the 3′ end of the genome. Black bars are shown as GMT of five hamsters in each group. Dotted line indicates the detection limit. Data were analyzed using two-way (*A*) or one-way (*B*–*M*) ANOVA (*P < 0.05; **P < 0.01; ***P < 0.001; ****P < 0.0001).

At week 7, hamsters in the rMeV, rMeV-S1, and rMeV-preS groups were moved to a biosafety level 3 (BSL3) animal facility and challenged intranasally with 10^5^ PFU of SARS-CoV-2. The normal control hamsters continued to be housed in the BSL2 animal facility and were inoculated with Dulbecco’s modified Eagle’s medium (DMEM). At day 4 postchallenge, five animals from each group were killed, and the remaining five animals were killed at day 12 postchallenge. We systemically evaluated the protection efficacy of rMeV-based vaccine candidates including clinical signs, weight loss, viral replication, RNA replication, cytokine responses in the lung, and lung histology and immunohistochemistry (IHC). Hamsters in the rMeV vector control group that were inoculated with SARS-CoV-2 exhibited clinical symptoms such as ruffled coat and weight loss (Fig. 5*A*). Hamsters in the rMeV group started to lose weight at day 1 postchallenge and reached ∼15% weight loss at day 6 and then started to regain weight from days 8 to 12 (Fig. 5*A*). Hamsters in the rMeV-S1 groups had similar weight loss from days 1 to 6 but had a faster weight recovery compared to the rMeV group (Fig. 5*A*). Importantly, hamsters in the rMeV-preS group did not have any abnormal reaction or weight loss. The body weight in the rMeV-preS group was not significantly different at most time points compared to the normal controls (Fig. 5*A*).

At day 4, five animals from each group were killed, and lungs, nasal turbinate, brain, liver, and spleen were collected for virus titration by plaque assay. An average titer of 7.4 × 10^5^ and 1.7 × 10^5^ PFU/g of SARS-CoV-2 were detected in lungs (Fig. 5*B*) and nasal turbinates (Fig. 5*C*) in the rMeV group, respectively. No infectious virus was detected in brain, liver, or spleen tissues in the rMeV group. Similarly, 4.4 × 10^5^ and 1.7 × 10^5^ PFU/g of SARS-CoV-2 were detected in lungs (Fig. 5*B*) and nasal turbinates (Fig. 5*C*) in the rMeV-S1 group, respectively, which were not significantly different from the rMeV group (*P* > 0.05). Importantly, infectious SARS-CoV-2 was below the detection limit in the lung in the rMeV-preS group (Fig. 5*B*) and only three out five animals had low viral titer (1.9 × 10^3^ PFU/g) in nasal tissue (Fig. 5*C*). At day 12, the remaining five hamsters in each group were killed. No infectious SARS-CoV-2 was detected in lung (Fig. 5*B*), nasal turbinate (Fig. 5*C*), or other tissues of any group.

To determine if SARS-CoV-2 genome RNA was present in these tissues we used primers annealing to the 5′ end of the SARS-CoV-2 genome. The highest number of background RNA copies detected in an unchallenged control group was set as the detection limit. As expected, high genome RNA copies were detected in both the lung (Fig. 5*D*) and nasal turbinate (Fig. 5*E*), moderate levels of viral RNA were detected in brain (Fig. 5*F*), and near-detectable levels of viral genome RNA were detected in liver (Fig. 5*G*) and spleen (Fig. 5*H*) tissues in the rMeV group at day 4. It should be noted that genomic RNA copies in lung, nasal turbinate, and brain in the rMeV-preS group were significantly lower than in the rMeV and rMeV-S1 groups (*P* < 0.001, *P* < 0.0001). Importantly, the average RNA copies in lungs, brain, liver, and spleen from the rMeV-preS group were near or below the detection limit whereas nasal turbinate had RNA titers of ∼10^4^ RNA copies/g tissue. At day 12, low levels of RNA were detected in nasal tissue and little or no RNA was detectable in all other tissues in all groups.

In addition to the full-length genome RNA, SARS-CoV-2 replication generates subgenomic RNA, which is more abundant than genomic RNA. Thus, we determined the levels of total viral RNA including genomic and subgenomic RNA using primers annealing to the N gene located at the 3′ end of the genome. Overall, the patterns of total RNA titers in lung (Fig. 5*I*), nasal turbinate (Fig. 5*J*), brain (Fig. 5*K*), liver (Fig. 5*L*), and spleen (Fig. 5*M*) were similar to those of genomic RNA in these tissue at days 4 and 12. Collectively, these results demonstrate that rMeV-preS vaccination provided complete protection against SARS-CoV-2 infection in hamsters whereas rMeV-S1 was unable to protect hamsters from SARS-CoV-2 infection.

### rMeV-preS Vaccination Prevents the SARS-CoV-2–Induced Cytokine Storm in Lungs.

**Fig. 6.**
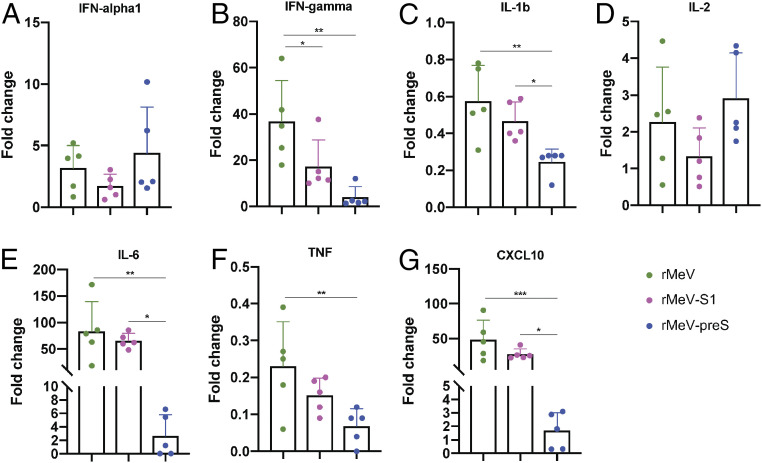
rMeV-preS immunization prevents a cytokine storm in the lungs. Total RNA was extracted from lungs of hamsters killed at day 4 after challenge with SARS-CoV-2. Hamster IFN-α1 (*A*), IFN-γ (*B*), IL-1b (*C*), IL-2 (*D*), IL-6 (*E*), TNF (*F*), and CXCL10 (*G*) mRNAs were quantified by real-time RT-PCR. GAPDH mRNA was used as an internal control. Data are shown as fold change in gene expression compared to normal animals (unimmunized and unchallenged) after normalization. Data were analyzed using one-way ANOVA (*P < 0.05; **P < 0.01; ***P < 0.001).

Cytokine storms play an important role in the pathogenesis and disease severity of COVID-19 patients (42). Thus, we determined whether rMeV-preS vaccination can prevent cytokine storm in the lungs. Briefly, IFN-α1, IFN-γ, IL-1b, IL-2, IL-6, TNF, and CXCL10 in lungs in each group were quantified by real-time RT-PCR and normalized to a control. Lung IFN-γ (Fig. 6*B*), IL-6 (Fig. 6*E*), and CXCL10 (Fig. 6*G*) mRNA had ∼17- to 36-, 66- to 84-, and 27- to 48-fold increases in rMeV and rMeV-S1 groups compared to the normal control group, respectively. However, the increases in these three cytokine mRNAs in the rMeV-preS group were minimal (two- to fourfold increase). Statistically, IFN-γ, IL-6, and CXCL10 were indistinguishable between the rMeV-preS group and the normal control group (*P* > 0.05). In addition, increases in TNF (Fig. 6*F*) and IL-1b (Fig. 6*C*) in the rMeV-preS group were significantly less than in rMeV and rMeV-S1 groups (*P* < 0.05). IFN-α1 (Fig. 6*A*) and IL-2 (Fig. 6*D*) in rMeV, rMeV-S, and rMeV-preS groups were similar (*P* > 0.05). These results suggest that rMeV-preS immunization prevents the cytokine storm in hamster lungs caused by a SARS-CoV-2 challenge.

### rMeV-preS Vaccination Protects Hamsters from SARS-CoV-2–Induced Lung Pathology.

**Fig. 7.**
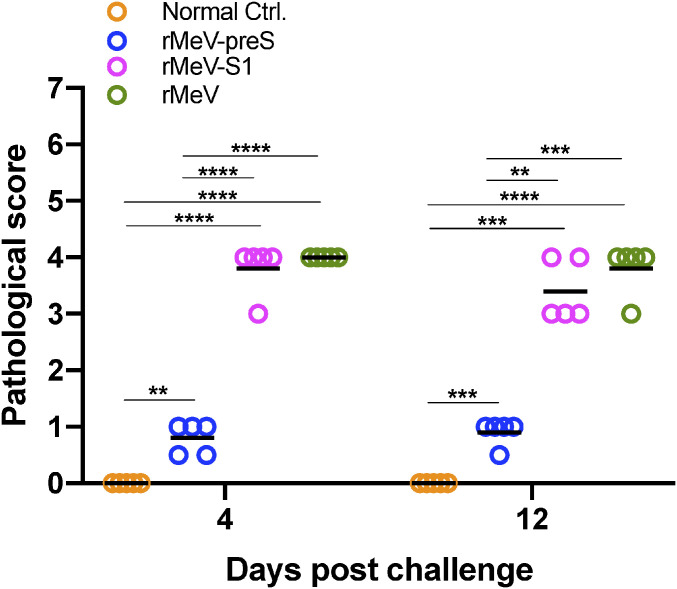
Lung pathology score after challenge with SARS-CoV-2. Fixed lung tissues from days 4 and 12 after SARS-CoV-2 challenge were embedded in paraffin, sectioned at 5 µm, deparaffinized, rehydrated, and stained with hematoxylin/eosin for the examination of histological changes by light microscopy. Each slide was quantified based on the severity of histologic changes including inflammation, interstitial pneumonia, edema, alveolitis, bronchiolitis, alveolar destruction, mononuclear cell infiltration, pulmonary hemorrhage, and peribronchiolar inflammation. Score 4 = extremely severe lung pathological changes; score 3 = severe lung pathological changes; score 2 = moderate lung pathological changes; score 1 = mild lung pathological changes; score 0 = no pathological changes. Data were analyzed using two-way ANOVA (**P < 0.01; ***P < 0.001; ****P < 0.0001).

**Fig. 8.**
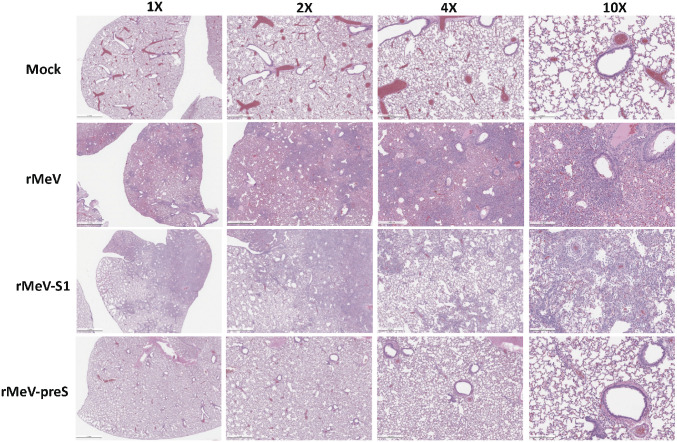
rMeV-preS immunization protects against lung pathology. Hematoxylin/eosin staining of lung tissue of hamsters killed at day 4 after SARS-CoV-2 challenge is shown. Micrographs with 1×, 2×, 4×, and 10× magnification of a representative lung section from each group are shown. Scale bars are indicated at the left corner of each image.

**Fig. 9.**
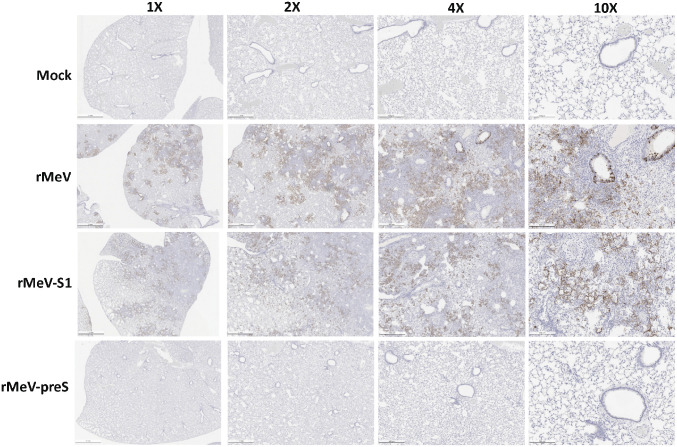
rMeV-preS immunization prevents SARS-CoV-2 antigen expression in lungs. IHC staining of lung sections from hamsters killed at day 4 after SARS-CoV-2 challenge is shown. Lung sections were stained with SARS-CoV-2 N antibody. Micrographs with 1×, 2×, 4×, and 10× magnification of a representative lung section from each group are shown. Scale bars are indicated at the left corner of each image.

All lungs from the hamster challenge study were stained with hematoxylin/eosin and the severity of histological changes was scored blindly by a trained veterinary pathologist (Fig. 7). At day 4 postchallenge, all lung tissues from the SARS-CoV-2–inoculated rMeV group had extremely severe lung histopathological changes (average score of 4.0) characterized by extensive inflammation, interstitial pneumonia, edema, alveolitis, bronchiolitis, alveolar destruction, mononuclear cell infiltration, pulmonary hemorrhage, and peribronchiolar inflammation (Figs. 7 and 8). Lung pathology in the rMeV-S1 group was also very severe (average score of 3.8) but slightly less than in the rMeV group (*P* > 0.05) (Figs. 7 and 8). In contrast, lung tissues from the rMeV-preS group had little to mild pathological changes (average score of 0.8) (Figs. 7 and 8). No lung pathology was found in the normal control group (score of 0) (Figs. 7 and 8). At day 12, lung pathology in the rMeV group was still extremely severe (average score of 3.8) (Fig. 7 and *SI Appendix*, Fig. S4). Severe lung pathology (average score of 3.4) was found in the rMeV-S1 group. However, mild lung pathology (score of 0.9) was detected in the rMeV-preS group (Fig. 7 and *SI Appendix*, Fig. S5). Lung sections were also stained with SARS-CoV-2 N antibody by IHC. At day 4, large amounts of SARS-CoV-2 N antigen were detected in all lung sections from the rMeV and rMeV-S1 groups (Fig. 9). In contrast, no N antigen was detected in lungs of the rMeV-preS group or the normal control (Fig. 9). At day 12, little N antigen was detected in the rMeV and rMeV-S1 groups and no antigen was detected in the lungs of the rMeV-preS group or normal control (*SI Appendix*, Fig. S5). These results demonstrate that rMeV-preS vaccination protects hamsters from lung pathology and prevents SARS-CoV-2 antigen expression in lungs.

## Discussion

In this study we developed a highly efficacious rMeV-based SARS-CoV-2 vaccine candidate. We found that the rMeV-preS–based vaccine candidate is more potent in triggering SARS-CoV-2–specific neutralizing antibody than the rMeV-based full-length S vaccine candidate. Antibodies induced by rMeV-preS were uniformly high in all four animal models including cotton rats, IFNAR^−/−^ mice, IFNAR1^−/−^-hCD46 mice, and Syrian golden hamsters and were significantly higher than antibody titers of human sera from convalescent COVID-19 patients. A single immunization of rMeV-preS was sufficient to induce a high level of SARS-CoV-2–specific antibody. In addition, rMeV-preS induces high levels of Th1-biased T cell immunity. Syrian golden hamsters immunized with rMeV-preS were completely protected against SARS-CoV-2 challenge including body weight loss, viral replication, cytokine storm, and lung pathology.

The MMR (measles, mumps, and rubella) vaccine is one of the most successful vaccines in human history (21, 22). Based on the Centers for Disease Control and Prevention data, one dose of MMR vaccine is 93% effective against MeV, 78% effective against mumps virus (MuV), and 97% effective against rubella. Two doses of MMR vaccine are 97% effective against MeV and 88% effective against MuV. Both MeV and MuV are nonsegmented negative-sense RNA virus and have potential as vectors to deliver foreign antigens. Particularly, MeV has been widely used as a vaccine vector. To date, more than 100 antigens have been expressed by MeV and more than 20 rMeV-based vaccines have been tested in preclinical trials (23, 43). Animal studies have shown that rMeV-based vaccines are highly effective against infectious diseases. Common immunization routes such as intramuscular, subcutaneous, intraperitoneal, and intranasal were effective to induce a high level of immune responses in cotton rats, IFNAR1^−/−^-hCD46 mice, and nonhuman primates (34, 44, 45). Currently, phase I clinical trials are being conducted to evaluate MeV-vectored vaccines against Zika virus (NCT02996890 and NCT04033068), Lassa virus (NCT04055454), and HIV (NCT01320176). In addition, a phase II clinical trials have demonstrated that a rMeV-vectored CHIKV vaccine was highly effective against CHIKV infection in humans (33).

Our data demonstrate that MeV is an excellent vaccine platform for delivering a SARS-CoV-2 vaccine. Live attenuated MeV vaccine has been widely used and has an excellent track record of high safety and efficacy in the human population since the 1960s (33, 46, 47). MeV grows to high titers in Vero cells, a WHO-approved cell line for vaccine production, facilitating vaccine manufacturing. Natural immunity to SARS-CoV-2 may not be long-lived (48, 49). However, MeV vaccine induces long-lasting immunity and protection against MeV infections (22, 50). By expressing the S protein from an rMeV vector, it may be possible to also induce long-lasting immunity to the S protein and to protect against COVID-19 disease. In areas of the world where MeV vaccination is not complete, such a combination vaccine could protect against both diseases. By incorporating rMeV-preS into the existing MMR vaccine, a quadruple vaccine could be developed against these four important pathogens for children. According to American Academy of Pediatrics, the number of US infants, children, and teens diagnosed with COVID-19 had reached more 2.6 million by 21 January 2021, accounting for 12.7% of all cases in the United States. Such a quadruple vaccine would be highly attractive for children.

In this study, we directly compared the efficacy of preS, native full-length S, S1, and three different lengths of RBD antigens in cotton rats. We found that the preS protein is the most potent antigen in inducing SARS-CoV-2–specific ELISA antibodies, but more importantly, neutralizing antibodies. All five cotton rats immunized with rMeV-preS triggered uniformly high antibody responses whereas antibody titers in the rMeV-S group were variable. Although there was no significant difference in antibody titers (*P* > 0.05), the rMeV-preS induced significantly higher neutralizing antibodies than rMeV-S (*P* < 0.05). Similarly, rMeV-preS induced uniformly high antibodies in other animal models including IFNAR^−/−^-CD46 mice and golden Syrian hamsters. In hamsters, the neutralizing antibody induced by rMeV-preS was significantly higher than human COVID-19 convalescent sera (*P* < 0.05). Our results suggest that preS is more immunogenic than native full-length S. It is likely that spontaneous triggering of the metastable native full-length S leads to release of the S1 portion of the protein and refolding to the postfusion form, thereby losing the prefusion-specific antigenic sites and reducing its ability to induce neutralizing antibodies. This is similar to many fusion glycoproteins (such as those from paramyxoviruses, pneumoviruses, and HIV) in that the “prefusion” form of proteins are more potent in inducing neutralizing activity than its “postfusion” forms (16–20). We also found that S1 and the RBDs are poor antigens in the MeV vector, which is probably due to the suboptimal conformation of these monomeric proteins. Interestingly, Pfizer’s BNT162b1, a lipid-nanoparticle-formulated, nucleoside-modified mRNA vaccine that encodes the trimerized RBD, was effective in triggering neutralizing antibody in human clinical trials (7, 51). Perhaps, the trimerization and/or adjuvants enhance its immunogenicity.

One important advantage of using rMeV-preS–based vaccine candidate is that rMeV-preS induced predominately a Th1-biased T cell response, thereby reducing the risk of potential antibody-dependent enhancement (ADE). We observed high frequencies of CD8^+^ T cells capable of producing Th1 cytokines, whereas frequencies of CD4^+^ T cells were low. Similar results were observed in an earlier study in which mice were vaccinated with recombinant adenovirus vector expressing SARS-CoV-2 S protein (52). Consistent with this, hamsters immunized with rMeV-preS were completely protected against SARS-CoV-2 challenge without any enhanced lung immunopathology. These results suggest that rMeV-preS is safe and highly efficacious. Historically, ADE has been a challenge in coronavirus vaccine development (53). It was reported that inactivated MERS-CoV vaccine candidates (54) and several SARS-CoV-1 vaccine candidates, including an inactivated whole-virus vaccine (55), virus-like-particle vaccine (56), and modified vaccinia virus Ankara-based recombinant vaccine (57), induced ADE in various animal models. Mechanistically, the excessive Th2-cytokine-biased responses and inadequate Th1-biased T cell response contributed to the immunopathology upon SARS-CoV-1 infection (53, 55, 56). Thus, an ideal SARS-CoV-2 vaccine should induce a high level of Th1 but not Th2-biased T cell response. Clearly, the rMeV-preS–based vaccine platform meets this criterion.

During preparation of this manuscript, Hörner et al. reported an rMeV-based SARS-CoV-2 vaccine candidate (58). They inserted the full-length S gene between the H and L genes, the last two genes of the MeV genome and therefore the S protein would be expressed at a lower level. A single immunization of this recombinant virus [MeV_vac2_-SARS2-S(H)] did not induce any SARS-CoV-2–specific neutralizing antibody. After booster immunization, only three out of seven animals produced detectable neutralizing antibody. After challenge with SARS-CoV-2, the MeV_vac2_-SARS2-S(H)-immunized hamsters had significant weight loss at postinfection days (PIDs) 1 to 3 but started to gain weight at PID 4. Furthermore, 4 to 5 log PFU/g tissue of SARS-CoV-2 were still detected in the nasal turbinate in MeV_vac2_-SARS2-S(H)–immunized hamsters. Thus, MeV_vac2_-SARS2-S(H) only induced partial protection against SARS-CoV-2 challenge (58). It should be noted that our study differs significantly from the Hörner’s study. We significantly improved the efficacy of the rMeV-based SARS-CoV-2 vaccine by using two different strategies. First, we generated rMeV expressing a stabilized, preS (rMeV-preS) and rMeV expressing full-length S protein (rMeV-S) and found that rMeV-preS was significantly more potent in inducing SARS-CoV-2–specific neutralizing antibodies than rMeV-preS. Second, our preS and S genes were inserted at the P and M gene junction, near the 3' end of the MeV genome. As a typical nonsegmented negative-sense RNA virus, MeV mRNA transcription is sequential and gradient such that 3′ proximal genes are transcribed more abundantly than 5′ distal genes, thereby producing more copies of their encoded proteins. Thus, the expression of preS and S in our vaccines is much higher than Hörner’s vaccine, which further enhance the immunogenicity. As shown in our report, rMeV-preS induced uniformly high levels of neutralizing antibody in all animals in all four animal models. A single immunization of rMeV-preS is sufficient to induce a high level antibody response. Importantly, rMeV-preS induced higher levels of neutralizing antibody than found in convalescent sera from COVID-19 patients. Furthermore, rMeV-preS provides complete protection against SARS-CoV-2 challenge.

In summary, we have developed a safe and highly efficacious rMeV-based preS vaccine candidate that can provide complete protection against severe SARS-CoV-2 infection and lung pathology in animal models, supporting its further development as a vaccine.

## Materials and Methods

Detailed descriptions of cell cultures, virus strains, construction of infectious cDNA clones of MeV, recovery and characterization of recombinant MeV expressing SARS-CoV-2 S proteins, multistep virus growth curves, preparation of large stock of rMeVs, MeV, and SARS-CoV-2 plaque assays, Western blot, RNA extraction, RT-PCR, RT-qPCR, human serum samples, animal studies in cotton rats, IFNAR1^−/−^ mice, IFNAR1^−/−^-hCD46 mice, and golden Syrian hamsters, purification of S protein, S peptides, T cell assay, ELISpot assay, quantification of intracellular cytokine production, flow cytometric analysis, detection of SARS-CoV-2–specific antibody by ELISA, detection of SARS-CoV-2 neutralizing antibody, determination of SARS-CoV-2 titer in hamster tissues, quantification of cytokine in lungs of hamsters, histology, IHC, and statistical analysis are provided in *SI Appendix*.

## Supplementary Material

Supplementary File

## Data Availability

All data are provided in the manuscript and *SI Appendix*. The GenBank accession number for the S gene of SARS-CoV-2 USA-WA1/2020 is MN985325.
