# Supplementary material for: A safe and highly efficacious measles virus-based vaccine expressing SARS-CoV-2 stabilized prefusion spike
Source: Proc Natl Acad Sci U S A. 2021 Mar 9;118(12):e2026153118. doi: 10.1073/pnas.2026153118 (PMC8000430; doi:10.1073/pnas.2026153118)
Supplement: Supplementary File [file pnas.2026153118.sapp.pdf]

PNAS
www.pnas.org

Supplementary Information for "A safe and highly efficacious measles virus-based vaccine expressing SARS-CoV-2 stabilized prefusion spike"

Mijia Lu, Piyush Dravid, Yuexiu Zhang, Sheetal Trivedi, Anzhong Li, Olivia Harder, Mahesh KC, Supranee Chaiwatpongsakorn, Ashley Zani, Adam Kenney, Cong Zeng, Chuanxi Cai, Chengjin Ye, Xueya Liang, Masako Shimamura, Shan-Lu Liu, Asuncion Mejias, Octavio Ramilo, Prosper N. Boyaka, Jianming Qiu, Luis Martinez-Sobrido, Jacob S. Yount, Mark E. Peeples, Amit Kapoor, Stefan Niewiesk, **Jianrong Li***

*Corresponding author
Department of Veterinary Biosciences
College of Veterinary Medicine
The Ohio State University

1925 Coffey Road
Columbus, OH 43210
Email: li.926@osu.edu

This PDF file includes:

Supplementary Materials and Methods
Figures S1 to S5
Tables S1 to S2
SI References

Supplementary Materials and Methods

Biosafety. All experiments with infectious SARS-CoV-2 were conducted under biosafety level 3 (BSL3) at The Ohio State University and were approved by the Institutional Biosafety Committee (IBC).

Cell cultures. Vero CCL81 cells (African green monkey, ATCC no. CCL81), Vero E6 cells (ATCC CRL-1586), and HEp-2 cells (ATCC no. CCL-23) were grown in Dulbecco's modified Eagle's medium (DMEM; Life Technologies) supplemented with 10% fetal bovine serum (FBS). FreeStyle293F cells (Thermo Fisher) were grown in protein-free medium in suspension culture.

Virus strain. The SARS-CoV-2 USA-WA1/2020 natural isolate (GenBank accession no. MN985325) was obtained from BEI Resources (NR-52281) and amplified on Vero E6 cells. This strain was originally isolated from an oropharyngeal swab from a patient with respiratory illness in January 2020 in Washington, USA.

Animals. Specific-pathogen-free (SPF) IFNAR1$^{-/-}$ and C57BL/6J-hCD46 mice were purchased from Jackson Laboratories (Bar Harbor, ME). Golden Syrian hamsters and cotton rats (*Sigmodon hispidus*) were purchased from Envigo (Indianapolis, IN). IFNAR1$^{-/-}$-hCD46/mice were generated by hybridization of IFNAR1$^{-/-}$ mice (Jackson laboratory) with C57BL/6J-hCD46 mice (Jackson laboratory). IFNAR1 knockout homozygous with hCD46 knock-in mice are derived by sib mating of the first filial generation. Genotype of IFNAR1$^{-/-}$ and hCD46 was determined by PCR from alkaline lysed ear tissue of each mouse. Sequences of PCR primers are: IFNAR1 common forward:

5'- CGA GGC GAA GTG GTT AAA AG; IFNAR1 wild type reverse: 5'- ACG GAT CAA CCT CAT TCC AC; IFNAR1 mutant reverse: 5'- AAT TCG CCA ATG ACA AGA CG; CD46 forward: 5'-GCC TGT GAG GAG CCA CCA A; CD46 reverse: 5'- CGT CAT CTG AGA CAG GTA G. For PCR reaction, 2 μl of mouse DNA was mixed with primers and 2× KAPA2G Fast HotStart Genotyping Mix with dye [KAPABIOSYSTEMS, KK5621 07961316001 (6.25 ml)].

Rapid assembly of the full-length genomic cDNA of MeV by yeast-based recombination system. The full-length genomic cDNA of Edmonston strain of measles vaccine was assembled into pYES2 vector. The pYES2 vector was modified to insert a yeast replication origin from the plasmid pYES1L (Invitrogen), a T7 RNA polymerase promoter, a hepatitis delta virus ribozyme (HDVRz) sequence, and a T7 terminator (1, 2). The full-length cDNA clone of MeV was constructed using six overlapping fragments (designated from A to F) by using yeast recombination system. Briefly, 100 ng of pYES2 vector was mixed with 200 ng of each MeV DNA fragment in PEG/LiAc solution, and the ligation products were transformed into MaV 203 competent yeast cells by heat-shock and plated on SD/Ura$^-$ agar plates. After incubation for 3 days at 30℃, individual colony was picked, cultured in SD/Ura$^-$ broth at 30℃ overnight for plasmid mini-prep. For initial screening, the connection regions between fragments were amplified by PCR and sequenced. The positive plasmid was then transformed into TOP10 competent cells, and plasmid DNA was verified by restriction enzyme digestion, PCR analysis, and sequenced to confirm that no additional mutations were introduced during the assembly. The final plasmid was designated as pMeV-SARS-CoV-2 (**Fig.S1**). Primers used in this study was listed in **Table S1.** Using this method, SARS-CoV-2 full-length S (S), a stabilized prefusion S (preS) with deletion of the furin cleavage site, two proline mutations, and a foldon trimerization domain (3), S with deletion of the

transmembrane domain and cytoplasmic tail (S-dTM), S1 subunit, and three different length of RBDs (RBD1, RBD2, and RBD3) containing MeV gene start and gene end sequences were inserting into the gene junction between P and M genes in the MeV genome. These plasmids were named pYES2-S, preS, S-dTM, S1, RBD1, RBD2, and RBD3. All the constructs were confirmed by sequencing. All the S genes and S truncations used in this study were codon optimized for mammalian cells expression.

Recovery of recombinant MeV (rMeV) expressing SARS-CoV-2 S antigens. Recovery of rMeV from the infectious clone was carried out as described previously (2, 4). Briefly, plasmid encoding the full-length genome of MeV Edmonston strain with S, preS, S-dTM, S1, or RBDs, and support plasmids encoding MeV ribonucleocapsid complex (pN, pP, and pL) were co-transfected into HEp-2 cells infected with a recombinant modified vaccinia Ankara virus (MVA-T7) expressing T7 RNA polymerase (kindly provided by Dr. Bernard Moss) (5). At day 4 post-transfection, cells and supernatants were collected, and co-cultured with 90% confluent Vero CCL81 cells. At day 4, the recovered recombinant virus was further amplified in Vero CCL81 cells. Subsequently, the viruses were plaque purified as described previously (6, 7). Individual plaques were isolated, and seed stocks were amplified in Vero CCL81 cells. The viral titer was determined by a plaque assay performed in Vero CCL81 cells.

RT-PCR verification of SARS-CoV-2 gene. To characterize the insertion of SARS-CoV-2 genes, viral RNA was extracted from rMeVs by using a RNeasy minikit (Qiagen, Valencia, CA) according to the manufacturer's instructions. SARS-CoV-2 S, preS, S-dTM, S1, RBD1, RBD2, and RBD3 genes were amplified by a One Step RT-PCR kit (Qiagen) using primers annealing to

MeV P gene and MeV M gene. The amplified products were analyzed on 1% agarose gel electrophoresis and sequenced. Primers used for RT-PCR and sequencing of pYES2-SARS-CoV-2 are listed in **Table S1**.

Multi-step growth curves. Confluent monolayers of Vero CCL81 cells in 12-well-plates were infected with individual viruses at a multiplicity of infection (MOI) of 0.01. After 1 h of absorption, the inoculum was removed, the cells were washed twice with Dulbecco's modified Eagle's medium (DMEM), fresh DMEM (supplemented with 2% fetal bovine serum) was added, and the infected cells were incubated at 37°C. The cell culture fluid and cell lysates were harvested and combined at the indicated intervals, and virus titers were determined by plaque assay in Vero CCL81 cells.

MeV and SARS-CoV-2 plaque assays. MeV and SARS-CoV-2 plaque assay was performed on Vero CCL81 and Vero-E6 cells in 12-well plates, respectively. For MeV, confluent Vero CCL81 cells in 12-well plates were infected with serial dilutions of rMeV or rMeV expressing SARS-CoV-2 antigen in DMEM. Similar procedure was used for SARS-CoV-2 plaque assay. After absorption for 1 h at 37 °C, cells were washed three times with DMEM and overlaid with 2 ml of DMEM containing low-melting agarose (0.25% w/v). After incubation at 37°C for 4-5 days (MeV) or 2 days (SARS-CoV-2), cells were fixed with 4% paraformaldehyde for 2 h. The overlays were removed, and the plaques were visualized after staining by crystal violet. The diameter of plaques for each virus were measured using Image J Software.

Preparation of large stock of rMeVs. T150 flasks of Vero CCL81 cells were infected with individual rMeV at a MOI of 0.1. When extensive CPEs were observed at day 3 or 4, the supernatants were harvested and kept on ice. Cell pellets were subjected to three freeze-thaw cycles in 0.5 ml of fresh DMEM with 10% trehalose (8). The two portions of supernatants were combined and the virus titers were determined by plaque assay in Vero CCL81 cells.

Detection of SARS-CoV-2 S antigen by Western blot. Vero CCL-81 cells were infected with parental rMeV or rMeV expressing SARS-CoV-2 S antigens as described above. At the indicated times post-infection, cell culture medium was harvested and clarified at 5,000 g for 15 min. In the meantime, cells were lysed in RIPA buffer (Abcam, ab156034). Proteins were separated by 12% SDS-PAGE and transferred to a Hybond enhanced chemiluminescence nitrocellulose membrane (Amersham) in a Mini Trans-Blot electrophoretic transfer cell (Bio-Rad). The blot was probed with rabbit anti-SARS-CoV-2 S or RBD antibody at a dilution of 1:2,000, followed by horseradish peroxidase-conjugated goat anti-rabbit IgG secondary antibody (Santa Cruz) at a dilution of 1:5,000. The blot was developed with SuperSignal West Pico chemiluminescent substrate (Thermo Scientific) and exposed to Kodak BioMax MR film.

Human sera. Human serum samples were collected from six SARS-CoV-2 positive individuals once diagnosis of SARS-CoV-2 was confirmed (V1) and 30 days later (V2). All human studies were conducted in compliance with all relevant local, state, and federal regulations and were approved by the Institutional Review Board (IRB) Nationwide Children's Hospital, Columbus, OH.

Animal experiments: All animals were housed within ULAR facilities of The Ohio State University under approved Institutional Animal Care and Use Committee (IACUC) guidelines (protocol no. 2009A0183 and 2020A00000053). Each inoculation group was separately housed in rodent cages under animal biosafety level 2 (BSL-2 for rMeV) or BSL3 (for SARS-CoV-2) conditions.

Immunogenicity in cotton rats. Cotton rats (*Sigmodon hispidus*) are susceptible to MeV infection (9, 10). Forty-five 4-week-old specific-pathogen-free (SPF) cotton rats (Envigo, Indianapolis, IN) were randomly divided into 9 groups, with 5 cotton rats per group (n=5). Cotton rats in groups 1-9 were inoculated subcutaneously with PBS, 4×10^5 PFU of each of Edmonston vaccine strain (parental rMeV, rMeV-S, rMeV-preS, rMeV-S1, rMeV-RBD1, rMeV-RBD2, or rMeV-RBD3). Four weeks later, cotton rats were boosted with 2×10^6 PFU of each virus at the same immunization route. After inoculation, the animals were evaluated twice every day for any possible abnormal reaction. Blood samples were collected from each cotton rat at weeks 4, 6, and 8 by retro-orbital bleeding, and the serum was isolated for antibody detection.

Immunogenicity in IFNAR$^{-/-}$-hCD46 transgenic mice. IFNAR$^{-/-}$-hCD46 transgenic mice that are deficient for type I IFN receptor and transgenically express human CD46 (11, 12) were bred in-house under SPF conditions. Twenty-one four-week-old female IFNAR1$^{-/-}$-hCD46 mice were randomly divided into 4 groups (n=5, or 6). Mice in groups 1-3 were immunized with 8×10^5 PFU (half subcutaneous and half intranasal) of parental rMeV, rMeV-preS, or rMeV-S1. Mice in group 4 served as normal controls (unimmunized and unchallenged controls). Two weeks later, mice were boosted with 6×10^5 PFU of each virus (half subcutaneous and half intranasal). After

inoculation, the animals were evaluated twice every day for safety. Blood samples were collected from each mouse at weeks 3 by facial vein bleeding, and the serum was isolated for antibody detection. At week 3 post-immunization, spleens were isolated from each mouse for a T cell assay.

Comparison of single and booster immunization of rMeV-preS in IFNAR$^{-/-}$ mice. 4-week-old IFNAR$^{-/-}$ mice female IFNAR1$^{-/-}$ mice were randomly divided into 3 groups (*n*=5, or 6). Mice in groups 1 were immunized with 8×10^5 PFU of rMeV-preS (half subcutaneous and half intranasal). Mice in group 2 were immunized with 8×10^5 PFU of rMeV-preS (half subcutaneous and half intranasal) and were boosted at the same dose at the same route 4 weeks later. Mice in group 3 were immunized with 8×10^5 PFU of rMeV and served as controls. At weeks 7 and 8, blood samples were collected from each mouse by facial vein bleeding, and the serum was isolated for detection of S-specific antibody by ELISA.

Immunization and challenge experiment in Golden Syrian hamsters. We selected 2 vaccine candidates (rMeV-preS and rMeV-S1) for immunization and challenge experiments in Golden Syrian hamsters. Forty 4-week-old female Golden Syrian hamsters were initially housed in BSL2 animal facility and randomly divided into 4 groups (*n* =10). Group 1 received 8×10^5 PFU of rMeV-preS, Group 2 received 8×10^5 PFU of rMeV-S1, Group 3 received 8×10^5 PFU of parental rMeV, and Group 4 received PBS. Three weeks later, hamsters in each group were boosted with the respective rMeV strain. All immunizations were done by combination of subcutaneous and intranasal routes (4×10^5 PFU for subcutaneous and 4×10^5 PFU for intranasal inoculation). At weeks 2, 4, and 6 post-immunization, blood was collected from each hamster for antibody detection. At week 4 post-booster immunization, animals of groups 1-3 were transferred into BSL3

facility and challenged intranasally with 10^5 PFU of SARS-CoV-2. Hamsters in group 4 were inoculated with DMEM and served as unimmunized unchallenged controls. After challenge, clinical sign and body weight of each hamsters were monitored daily. At day 4 post-challenge, 5 hamsters in each group were euthanized, left lung, nasal turbinate, brain, liver, and spleen were collected for detection of SARS-CoV-2 and viral RNA. In addition, the right lung was preserved in 4% (vol/vol) phosphate-buffered formaldehyde for histology and immunohistochemistry (IHC). At day 12 post-challenge, the remaining 5 hamsters were terminated, and tissues were collected and processed as described above.

S protein purification. The stabilized prefusion S protein (amino acids 1-1273) of SARS-CoV-2 was cloned into pCAGGS and transfected into FreeStyle293F cells for protein expression. The secreted preS in cell culture supernatants were then purified via affinity chromatography. The purity of the protein was analyzed by SDS-PAGE and Coomassie blue staining. Protein concentration was measured using Bradford reagent (Sigma Chemical Co., St. Louis, MO).

Peptides. A set of 181 peptides spanning the complete S protein of the USA-WA1/2020 strain of SARS-CoV-2 (GenPept: QHO60594) were obtained from BEI resources (National Institute of Allergy and Infectious Diseases) (cat.no. NR-52402). These peptides are 13 to 17 amino acids long, with 10 amino acid overlaps. The Spike 1 (S1) peptide pools contain 93 peptides representing the N terminal half of the S protein (MFVFLVLLPL to AEHVNNSYE) and the Spike 2 (S2) peptide pools contain 88 peptides representing the C terminal half of the S protein (GAEHVNNSYE to VLKGVKLHYT). Peptides were dissolved in sterile water containing 10% DMSO. The final concentration of each peptide in all functional assays was 2 μg/ml.

ELISPOT assay. Spleens of immunized IFNAR$^{-/-}$-CD46 transgenic mice were aseptically removed 35 days after immunization and minced by pressing through cell strainers. Red blood cells were removed by incubation in 0.84 % ammonium chloride and, following a series of washes in RPMI 1640, cells were resuspended in RPMI 1640 supplemented with 2 mM l-glutamine, 1 mM sodium pyruvate, 10 mM HEPES, 100 U/ml penicillin, 100 µg/ml streptomycin, and 10% fetal calf serum. Antigen-specific T cells secreting IFN-γ were enumerated using anti-mouse IFNγ enzyme linked immunospot (ELISpot) assay (U-Cytech catalogue no. CT317-PB5). Cells were plated in 96 well PVDF plates at 2×10^5 per well in duplicate, and stimulated separately with the SARS-CoV-2 peptide pools (2 µg/ml), Concanavalin-A (5 µg/ml, Sigma) or media alone, as positive and negative controls, receptively. The plates were incubated for 42-48 h and then developed according to manufacturer's instructions. The number of spot-forming cells (SFC) were measured using an automatic counter (Immunospot). A positive response was considered only when the mean of peptides-stimulated wells was more than the mean of negative wells + 3 standard deviation. The total number of spot-forming cells (SFC) were calculated by subtracting the mean number of SFC in negative control wells from that of peptides containing wells.

Quantification of intracellular cytokine production. For detection of SARS-CoV-2-specific intracellular cytokine production, 10^6 cells were stimulated in 96-well round bottom plates with peptide pool (5 µg/ml), media alone or PMA/Ionomycin (BioLegend) as negative and positive controls, respectively, for 5-h in the presence of GolgiPlug (BD Biosciences). Following incubation, cells were surface stained for CD3, CD4, and CD8 for 30 min at 4°C, fixed and

permeabilized using the cytofix/cytoperm kit (BD Biosciences), and intracellularly stained for IFNγ, TNFα, IL-2, Granzyme B, IL-10 & IL-4 for 30 min at room temperature. Dead cells were removed using the LIVE/DEAD fixable Near-IR dead cell stain kit (Invitrogen). A positive response was defined as >3 times the background of the negative control sample. The percentage of cytokine positive cells was then calculated by subtracting the frequency of positive events in negative control samples from that of test samples.

Flow cytometric analysis. The following mouse reactive antibodies (clone, catalog number, dilution) from BioLegend, BD Biosciences, and ThermoFisher Scientific were used for analysis of T cells: CD3-PE/Cyanine7 (145-2C11, 100319, 1:400), IFNγ-PE/Dazzle 594 (XMG1.2, 505845, 1:400), TNFα-Brilliant Violet 785 (MP6-XT22, 506341, 1:400), CD107a-Alexa Fluor 488 (1D4B, 121607, 1:400), granzyme-B-Alexa Fluor 647 (GB11, 515405 1:200), IL-4-Brilliant Violet 711 (11B11, 504133, 1:100), CD4-BUV 496 (GK1.5, 612952, 1:400), CD8-BUV737 (53-6.7, 612759, 1:400), IL-10-Brilliant Violet 510 (JES5-16E3, 563277, 1:100), IL-2-PE (JES6-5H4, 12-7021-82, 1:200). Surface and intracellular staining was performed as described previously (13). Events were collected on a BD LSRFortessa X-20 flow cytometer following compensation with UltraComp eBeads (Invitrogen). Data were analyzed using FlowJo v10 (Tree Star).

Detection of SARS-CoV-2-specific antibody by ELISA. Ninety-six-well plates were first coated with 50 µl of highly purified prefusion SARS-CoV-2 preS protein (8 µg/ml, in 50 mM Na_2CO3 buffer, pH 9.6) per well at 4°C overnight, and then blocked with Bovine Serum Albumin (BSA, 1% W/V in PBS, 100 µl/well) at 37°C for 2 h. Subsequently, individual serum samples were tested for S-specific Ab on antigen-coated plates. Briefly, serum samples were 2-fold serially diluted and

added to S protein-coated wells (100 µl/well). After 2 h of incubation at room temperature, the plates were washed three times with phosphate-buffered saline containing 0.05% Tween (PBST), followed by incubation with 100 µl of horseradish peroxidase (HRP)-conjugated secondary Abs (Sigma) at a dilution of 1:15,000 for 1 h. The plates were washed, developed with 100 µl of SureBlue™ TMB 1-Component Microwell Peroxidase Substrate (Fisher Scientific, Catalog No.50-674-93), and stopped by 100 µl of H_2SO_4 (2 mol/L). Optical densities (OD) at 450 nm were determined by a BioTek microplate reader. Endpoint titers were determined as the reciprocal of the highest dilution that had an absorbance value 2.1 folds greater than the background level (normal control serum). Ab titers are reported as geometric mean titers (GMT).

Detection of SARS-CoV-2 neutralizing antibody by plaque reduction. SARS-CoV-2-specific neutralizing antibody was determined using an endpoint dilution plaque reduction neutralization (PRNT) assay. The serum samples were heat inactivated at 56°C for 30 min. Two-fold dilutions of the serum samples were mixed with an equal volume of DMEM containing approximately 100 PFU/well SARS-CoV-2 in a 96-well plate, and the plate was incubated at 37°C for 1 h with constant rotation. The mixtures were then transferred to confluent Vero-E6 cells in a 12-well plate. After 1 h of incubation at 37°C, the virus-serum mixtures were removed and the cell monolayers were covered with 1 ml of Eagle's minimal essential media (MEM) containing 0.25% agarose, 0.12% sodium bicarbonate (NaHCO3), 2% FBS, 25mM HEPES, 2mM L-Glutamine, 100µg/ml of streptomycin, and 100U/ml penicillin. Then, the cells were incubated for another 2 days and then fixed with 4% formaldehyde. The plaques were counted; serum dilution with 50% plaque reduction were calculated as the SARS-CoV-2-specific neutralizing antibody titers.

Determination of SARS-CoV-2 titer in hamster tissues. After SARS-CoV-2 challenge, left lung, nasal turbinate, brain, liver, and spleen was collected. Organs were weighed and homogenized by hand with a mortar and pestle (Golden, CO) in 1mL of sterile PBS. Each sample was subjected to 10-fold serial dilutions. The initial dilution of each tissue sample is 1:10. The presence of infectious SARS-CoV-2 was determined by plaque assay in Vero-E6 cells in 12-well plates. The limit of detection (LoD) is calculated with the following formula: LoD = Log_{10} [1(1 plaque in a well) / 0.2 (0.2ml tissue sample) ×10 (lowest dilution) / average tissue weight].

Measurement of SARS-CoV-2 genomic and subgenomic RNA burden. The total RNA was extracted from homogenized left lung, nasal turbinate, brain, liver, and spleen tissue samples using TRIzol Reagent (Life technologies, Carlsbad, CA). For total viral RNA (genome and subgenome), reverse transcription (RT) was conducted using a primer (5′-GTCATTCTCCTAAGAAGCTATTAAAATC-3′) targeting the 3'-UTR of SARS-CoV-2 and the Superscript III transcriptase kit (Invitrogen, Carlsbad, CA). For genome RNA, the RT primer (5'-GTGTCTTTGATTTCGAGCAAC-3') was annealing to 5' of SARS-CoV-2 genome. The RT products were then used to perform real-time PCR using primers specifically targeting the N gene of SARS-CoV-2 (forward, 5′- CATTGGCATGGAAGTCACAC -3′; reverse, 5′-TCTGCGGTAAGGCTTGAGTT -3′) or targeting the 5'-end of SARS-CoV-2 genome (forward, 5′- ACTGTCGTTGACAGGACACG -3′; reverse, 5′- ACGTCGCGAACCTGTAAAAC -3′) in a StepOne real-time PCR system (Applied Biosystems). A standard curve was generated using a plasmid encoding the nucleocapsid (N) gene or full-length genome of SARS-CoV-2 plasmid. Amplification cycles used were 2 min at 95°C, and 40 cycles of 15 s at 95°C, and 1 min at 60°C. The threshold for detection of fluorescence above the background was set within the exponential

phase of the amplification curves. For each assay, 10-fold dilutions of standard plasmid or viral RNA were generated, and negative-control samples and double-distilled water (ddH_2O) were included in each assay. After real-time qPCR, the Ct value from each sample was converted into $\log_{10}$ viral RNA copies/mg tissue according to the standard curve. The RNA copies were calculated with the following formula: RNA copies/mg tissue = Log_{10} [Ct-converted copies/µl×10(2µl from 20µl total cDNA) ×25 (2µl from 50µl total RNA) ×10 (100µl from 1ml homogenized tissue) / tissue weight (mg)]. The LoD is set as the maximum value of the normal control group. The exact $\log_{10}$ RNA copies/mg was reported for each sample.

Quantification of cytokine in lungs of hamsters. Total RNA was extracted from lungs of Golden Syrian hamsters, and IFN-α1, IFN-γ, IL-1b, IL-2, IL-6, TNF, and CXCL10 mRNAs were quantified by real-time RT-PCR (14, 15). GAPDH mRNA was used as internal controls. The cytokine mRNA of each group was expressed as fold-change in gene expression compared to normal animals (unimmunized and unchallenged) after normalization. Primers used for RT-qPCR were listed **in Table S2**.

Histology. The right lung lobes from each hamster were preserved in 4% (vol/vol) phosphate-buffered paraformaldehyde for 14 days before transferred out of the BSL-3 facility. Fixed tissues were embedded in paraffin, sectioned at 5 µm, deparaffinized, rehydrated, and stained with hematoxylin-eosin (HE) for the examination of histological changes by light microscopy.

Immunohistochemistry (IHC). Five-micron sections of paraffin embedded tissues were placed onto positively charged slides. After deparaffinization, sections were incubated with target

retrieval solution (Dako, Carpinteria, CA) for antigen retrieval. After blocking, lung sections were subjected to IHC staining using a rabbit SARS-CoV-2 N protein (NB100-56576, Novus Biologicals). Slides were counter stained with hematoxylin.

Statistical analysis. Quantitative analysis was performed by either densitometric scanning of autoradiographs or by using a phosphorimager (Typhoon; GE Healthcare, Piscataway, NJ) and ImageQuant TL software (GE Healthcare, Piscataway, NJ) or Image J software (NIH, Bethesda, MD). Statistical analysis was performed by one-way or two-way multiple comparisons using SPSS (version 8.0) statistical analysis software (SPSS Inc., Chicago, IL), two-way ANOVA, or Student's *t* test. A *P* value of <0.05 was considered statistically significant.

References

1. Y. J. Lu *et al.*, Porcine Epidemic Diarrhea Virus Deficient in RNA Cap Guanine-N-7 Methylation Is Attenuated and Induces Higher Type I and III Interferon Responses. *J Virol* **94** (2020).
2. Y. Wang *et al.*, Enhancement of safety and immunogenicity of the Chinese Hu191 measles virus vaccine by alteration of the S-adenosylmethionine (SAM) binding site in the large polymerase protein. *Virology* **518**, 210-220 (2018).
3. D. Wrapp *et al.*, Cryo-EM structure of the 2019-nCoV spike in the prefusion conformation. *Science* **367**, 1260-1263 (2020).
4. F. Radecke *et al.*, Rescue of Measles Viruses from Cloned DNA. *Embo J* **14**, 5773-5784 (1995).
5. T. R. Fuerst, E. G. Niles, F. W. Studier, B. Moss, Eukaryotic Transient-Expression System Based on Recombinant Vaccinia Virus That Synthesizes Bacteriophage-T7 Rna-Polymerase. *P Natl Acad Sci USA* **83**, 8122-8126 (1986).
6. J. R. Li, J. T. Wang, S. P. J. Whelan, A unique strategy for mRNA cap methylation used by vesicular stomatitis virus. *P Natl Acad Sci USA* **103**, 8493-8498 (2006).
7. J. R. Li, E. C. Fontaine-Rodriguez, S. P. J. Whelan, Amino acid residues within conserved domain VI of the vesicular stomatitis virus large polymerase protein essential for mRNA cap methyltransferase activity. *J Virol* **79**, 13373-13384 (2005).
8. M. G. Xue *et al.*, Stable Attenuation of Human Respiratory Syncytial Virus for Live Vaccines by Deletion and Insertion of Amino Acids in the Hinge Region between the mRNA Capping and Methyltransferase Domains of the Large Polymerase Protein. *J Virol* **94** (2020).

9. M. G. Green, D. Huey, S. Niewiesk, The cotton rat (Sigmodon hispidus) as an animal model for respiratory tract infections with human pathogens. *Lab Animal* **42**, 170-176 (2013).
10. S. Niewiesk, Current animal models: cotton rat animal model. *Curr Top Microbiol Immunol* **330**, 89-110 (2009).
11. C. Nurnberger, B. S. Bodmer, A. H. Fiedler, G. Gabriel, M. D. Muhlebach, A Measles Virus-Based Vaccine Candidate Mediates Protection against Zika Virus in an Allogeneic Mouse Pregnancy Model. *J Virol* **93** (2019).
12. M. Mura *et al.*, hCD46 receptor is not required for measles vaccine Schwarz strain replication in vivo: Type-I IFN is the species barrier in mice. *Virology* **524**, 151-159 (2018).
13. A. S. Hartlage *et al.*, Vaccination to prevent T cell subversion can protect against persistent hepacivirus infection. *Nat Commun* **10** (2019).
14. M. Zivcec, D. Safronetz, E. Haddock, H. Feldmann, H. Ebihara, Validation of assays to monitor immune responses in the Syrian golden hamster (Mesocricetus auratus). *J Immunol Methods* **368**, 24-35 (2011).
15. D. Safronetz *et al.*, Pathogenesis and host response in Syrian hamsters following intranasal infection with Andes virus. *PLoS Pathog* **7**, e1002426 (2011).

Supplementary Figures

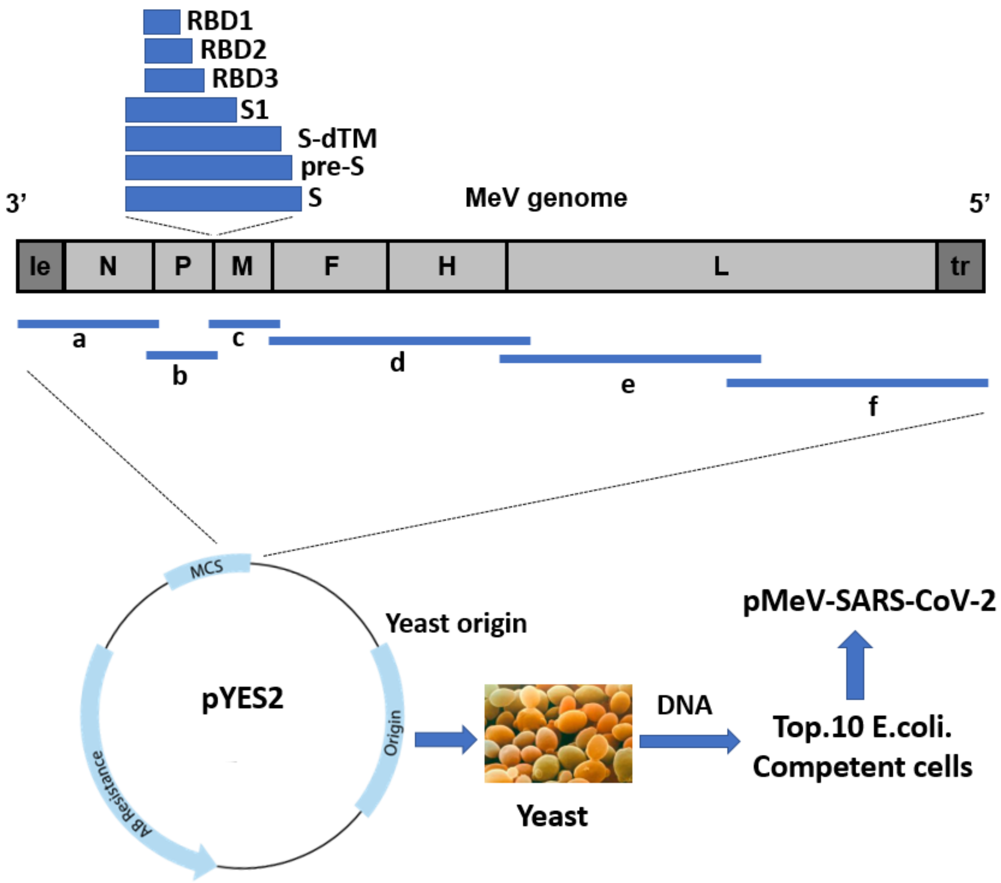

Fig.S1. A rapid method for construction of recombinant measles virus (rMeV) expressing SARS-CoV-2 antigens. **Top panel**: The SARS-CoV-2 S, preS, S-dTM, S1, RBD1, RBD2, and RBD3 were amplified from a codon optimized S gene of SARS-CoV-2 by PCR, and inserted at the gene junction between P and M in the genome of measles virus (MeV) Edmonston vaccine strain. **Middle panel**: The organization of negative-sense measles virus genome is shown. Le, leader sequence; N, nucleocapsid gene; P, phosphoprotein gene; M, matrix protein gene; F, fusion protein gene; H, hemagglutinin protein gene; L, large polymerase gene; Tr, trailer sequence. The plasmid pYES2 vector was modified to insert a yeast replication origin, a T7 RNA polymerase promoter, a hepatitis delta virus (HDV) ribozyme sequence, and a T7 terminator. The full-length cDNA clone of MeV with SARS-CoV-2 S gene was constructed using six overlapping fragments (designated from a to f) by using DNA recombinase in yeasts. **Low panel**: The map of plasmid pYES2 containing the yeast origin is shown. After recombination in yeast, DNA was extracted and transformed into Top.10 E. coli. competent cells. Plasmid pYES2 expressing SARS-CoV-2 S, preS, S-dTM, S1, RBD1, RBD2, and RBD3 was constructed.

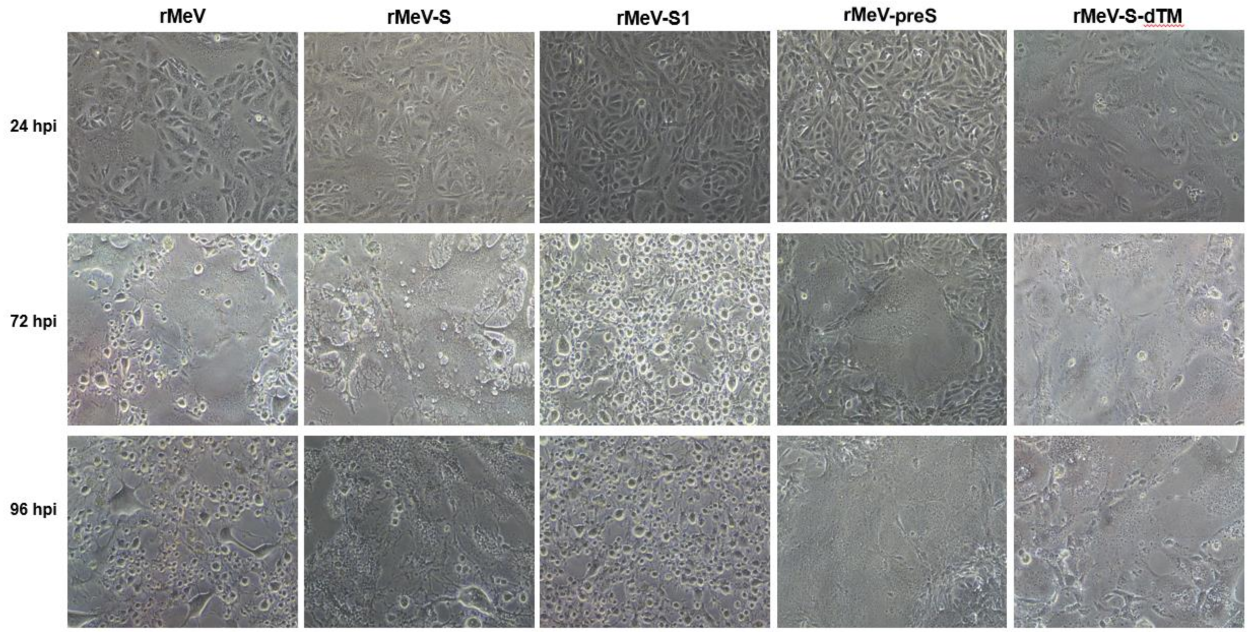

Fig.S2. **Recombinant MeV expressing SARS-CoV-2 antigens exhibit delayed syncytia formation and cytopathic effects (CPE).** Confluent Vero CCL81 cells in 12-well-plates were infected with individual virus at an MOI of 0.01. After 1 h of absorption, fresh DMEM with 2% FBS was added. Representative images of syncytia and CPE from each virus-infected cells were captured by light microscope at the indicated time points. Images are the representatives of three independent experiments.

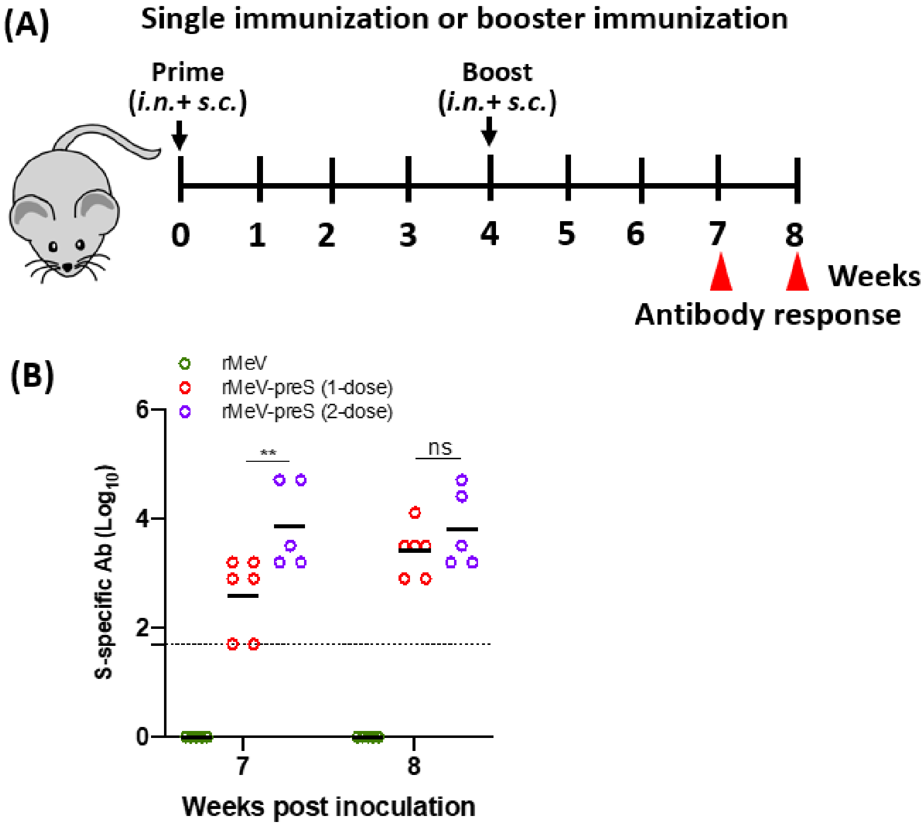

Fig.S3. A single immunization of rMeV-preS induces a strong antibody response in IFNAR$^{-/-}$ mice. (A) Immunization schedule. 4-week-old IFNAR$^{-/-}$ mice female IFNAR1$^{-/-}$ mice were randomly divided into 3 groups (*n*=5, or 6). Mice in groups 1 were immunized with 8×10^5 PFU of rMeV-preS (half subcutaneous and half intranasal). Mice in group 2 were immunized with 8×10^5 PFU of rMeV-preS (half subcutaneous and half intranasal) and were boosted at the same dose at the same route 4 weeks later. Mice in group 3 were immunized with 8×10^5 PFU of rMeV and served as controls. At weeks 7 and 8, blood samples were collected from each mouse by facial vein bleeding, and the serum was isolated for detection of S-specific antibody by ELISA. (B) Measurement of SARS-CoV-2 S-specific antibody by ELISA. Highly purified preS protein was used as the coating antigen for the ELISA. Dot line indicates the detectable level at the lowest dilution. Data are expressed as the geometric mean titers (GMT) of 5 or 6 mice ± standard deviation. Data were analyzed using Student's *t*-test ($**P < 0.01$; ns indicates no significant difference).

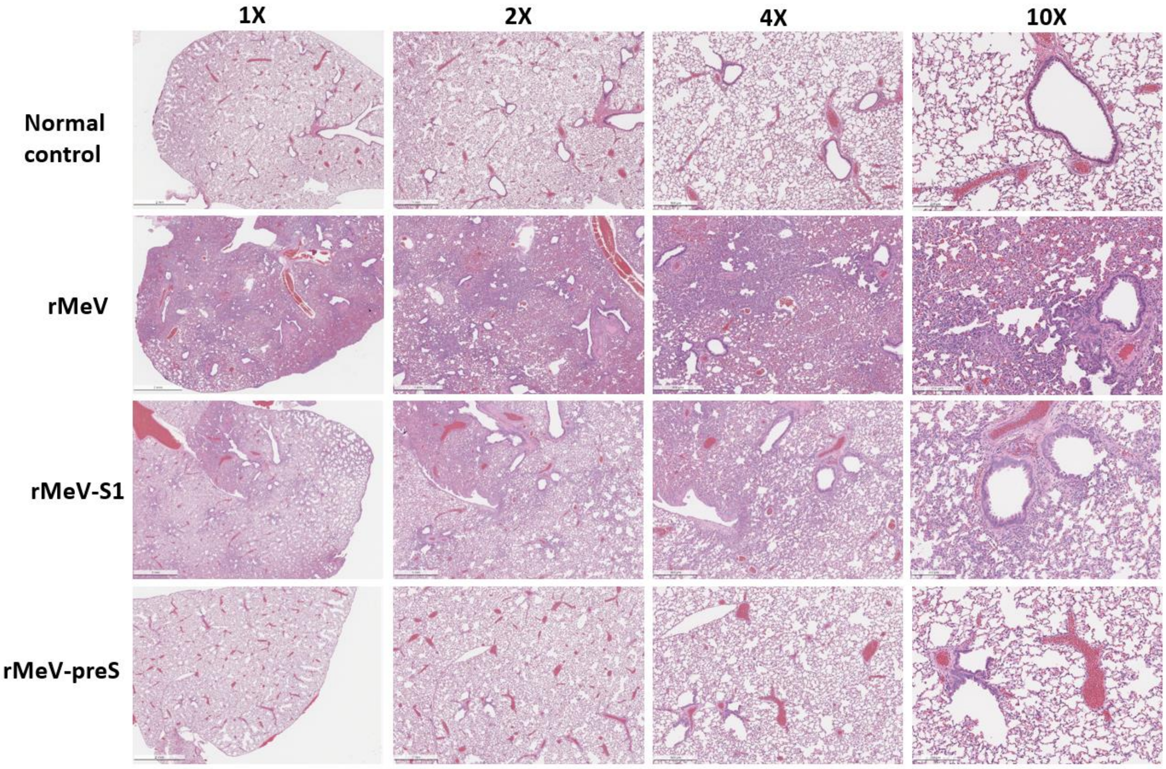

Fig.S4. **Histology of lung sections at day 12.** Hematoxylin-eosin (HE) staining of lung tissue of hamsters euthanized at day 12 after SARS-CoV-2 challenge is shown. Micrographs with ×1, ×2, ×4, and ×10 magnification of representative lung section are shown. Scale bars are indicated at the left corner of each image.

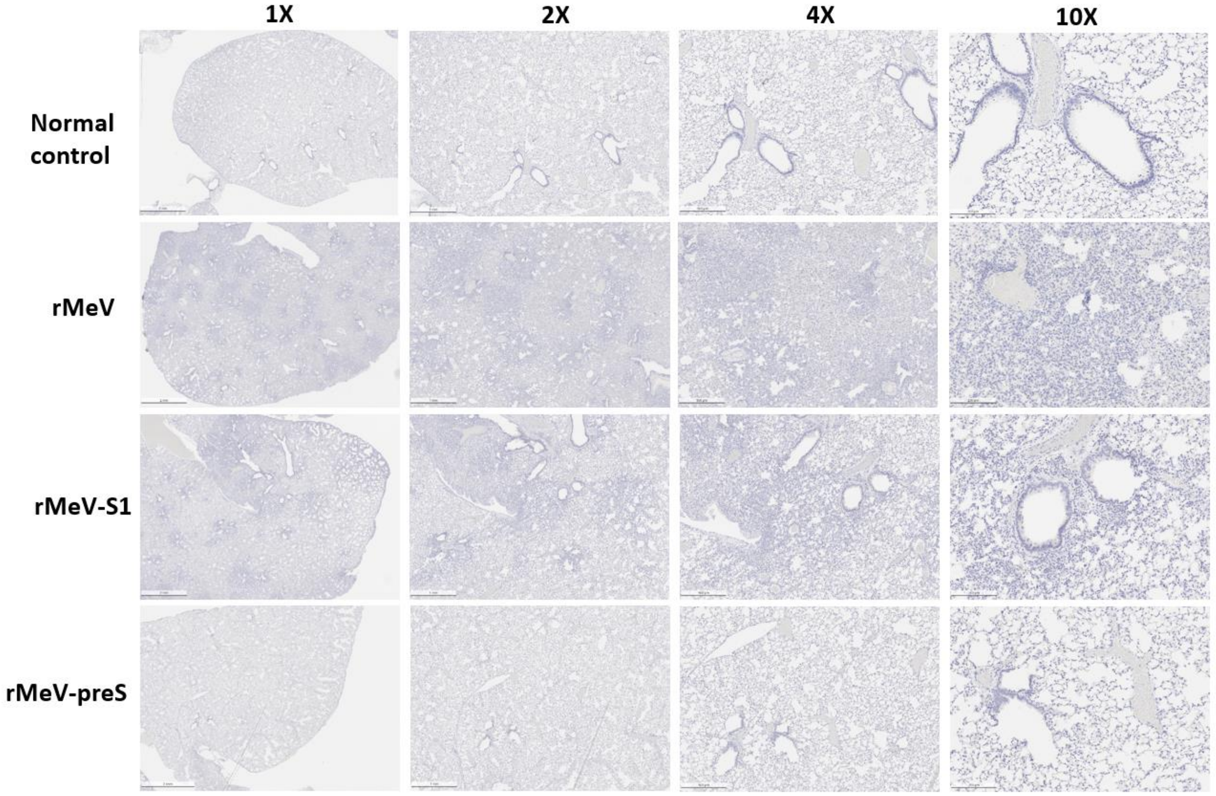

Fig.S5. **Immunohistochemistry (IHC) staining of lung sections at day 12.** IHC analysis of lung sections from hamsters euthanized at day 12 after SARS-CoV-2 challenge is shown. Lung sections were stained with SARS-CoV-2 N antibody. Micrographs with ×1, ×2, ×4, and ×10 magnification of representative lung section are shown. Scale bars are indicated at the left corner of each image.

Supplementary Table 1: Primers used in construction of infectious cDNA clones expressing SARS-CoV-2 S, preS, S-dTM, S1, RBD1, RBD2, and RBD3 genes

**Name**	**Sequences (5' to 3')**
Primers used for cloning SARS-CoV-2 S genes	
S/S1/S-dTM opti F	CGCCAGCCCATCAACGCGTATAATGTTTGTTTTCCTGGTGCTGCTGC
S opti R	TTTATAATGGATTTAGGTTGTATCATGTATAGTGGAGCTTGACGCCCTTC
S1 opti R	TTTATAATGGATTTAGGTTGTATTATCAGGACATTGTATAAGCAATAATGGAC
S opti dTM (1208) R:	TTTATAATGGATTTAGGTTGTAttatcaCTGCTCGTACTTGCCGAGCTCCTGAAG
RBD opti short F: (CD5 SP)	ATACTCGAGATGCCCATGGGGTCTCTGCAACCGCTGGCCACCTTGTACCTGCTGGGAA TGCTGGTTGCTTCCTGCCTCGGAAACATCACAAACCTGTGTCCCTTCG
RBD opti medium/long F: (CD5 SP)	ATACTCGAGATGCCCATGGGGTCTCTGCAACCGCTGGCCACCTTGTACCTGCTGGGAA TGCTGGTTGCTTCCTGCCTCGGACGCGTCCAGCCAACCGAGTCCATTG
RBD opti short R:	TTTATAATGGATTTAGGTTGTATTAGACGGTAGCTGGAGCGTGCAGCAGC
RBD opti medium R:	TTTATAATGGATTTAGGTTGTATTATCAGAAATTCACGCATTTGTTCTTG
RBD opti long R:	TTTATAATGGATTTAGGTTGTAttatcaCGGAGTAATGTCAAGGATTTCCAATG
RBD opti-P F:	CGCCAGCCCATCAACGCGTATAGAGATGCCCATGGGGTCTCTGCAACCGC
preS-F	CGCCAGCCCATCAACGCGTATAATGTTCGTGTTCCTGGTGCTCCTGC
preS-R	TTTATAATGGATTTAGGTTGTATCATTAGCCCAGGAATGTGCTCAGCAGTAC
Primers used for construction of infectious cDNA clone of MeV	
MeV-5end-F	ACCAAACAAAGTTGGGTAAGGATA
MeV-N-R	GGTAGGCGGATGTTGTTCTG
MeV-P-F	CTTCTAGACTAGGTGCGA
MeV-P (S opti) R	GCAGCAGCACCAGGAAAACAAACATTATACGCGTTGATGGGCTGGCG
MeV-P (CD5) R:	GCGGTTGCAGAGACCCCATGGGCATGAGTATACGCGTTGATGGGCTGGCGGCT
MeV-P (preS) R:	GCAGGAGCACCAGGAACACGAACATTATACGCGTTGATGGGCTGGCG
MeV-M (S opti) F	GAAGGGCGTCAAGCTCCACTATACATGATACAACCTAAATCCATTATAAA
MeV-M (S1 opti) F	GTCCATTATTGCTTATACAATGTCCTGATAATACAACCTAAATCCATTATAAA
MeV-M (RBD opti short) F	GCTGCTGCACGCTCCAGCTACCGTCTAATACAACCTAAATCCATTATAAA
MeV-M (RBD opti medium) F	CAAGAACAAATGCGTGAATTTCTGATAATACAACCTAAATCCATTATAAA
MeV-M (RBD opti long) F	CATTGGAAATCCTTGACATTACTCCGtgataaTACAACCTAAATCCATTATAAA
MeV-M (S opit dTM) F	CTTCAGGAGCTCGGCAAGTACGAGCAGtgataaTACAACCTAAATCCATTATAAA
MeV-M (preS) F	GTACTGCTGAGCACATTCCTGGGCTAATGATACAACCTAAATCCATTATAAA
MeV-M-R	CATGAATATGGCAGAGACGT
MeV-F-F	CCCGACGACACTCAACTCCC
MeV-H-R	ACGTTTTTCTTAATTCTGATGTCTAT
MeV-L1-F	ACATCAGGCATACCCACTA
MeV-L1-R	CCCACATATGGCTTCTTAG
MeV-L2-F	GACAAAGAGTCATGTTCAGTG
MeV-3end-R	CAGACAAAGCTGGGAATAG
pYES2-F	GCAATATATTAAAGAAAACTTTGAAAATACG
MeV-5end-R	GCACTAGAAGATGATCATTGATTGAAC
Primers used for junction PCR:	
MeV-F-R	CCTAAGTTTTAATTAACTACCGATA
MeV-H-F	TCCCTCTGGCCGAACAAT
M7281F	GCAACCAAACCAGAACCCAGACCAC
L1R11430	CTAGGTGAACTTCAGGGTATAAGATCTGGT
L1F14034	CGTTACCTTGCATATTCCCTGAACG
L2R15531	GGGAGCTGGGTATCCTGGGATGATCTATC
S opti sq R93	GTACGCAGGGGGCAGCTGGG

Supplementary Table 2: Primers used for RT-qPCR of hamster cytokines

Cytokine	GenBank Accession No.	Primer name and sequence
IFN-α1	S78750.1	Ham-IFN-a1-F: GCCTCTACCAGCAGCTCAGT<br>Ham-IFN-a1-R: GCAGACAGGGTTCTCCAGAC
IFN-γ	AF034482.1	Ham-IFN-g-F: CCATCAAGGCAGACCTGTTT<br>Ham-IFN-g-R: TTCTTGTTGGGACGATTTCC
IL-1β	AB028497.1	Ham-IL-1b-F: GGTGGTGTCAGTCATTGTGG<br>Ham-IL-1b-R: AGACAGCACGAGGCATTTCT
IL-2	EU729351.1	Ham-IL-2-F: TGCACCCACTTCAAGCTCTA<br>Ham-IL-2-R: GCCTTCTTGGGCATGTAAAA
IL-6	AB028635.1	Ham-IL-6-F: CTCCGCAAGAGACTTCCATC<br>Ham-IL-6-R: ACCAAACCTCCGACTTGTTG
TNF	XM_005086799.3	Ham-TNF-F: GACGGGCTGTACCTGGTTTA<br>Ham-TNF-R: GAGTCGGTCACCTTTCTCCA
CXCL10	NM_001281344.1	Ham-CXCL10-F: TGCCAGTGCTATCCTCCTTT<br>Ham-CXCL10-R: GATGGCCTCAGACTCTGGAT
GAPDH	DQ403055.1	Ham-GAPDH-F: AACTTTGGCATTGTGGAAGG<br>Ham-GAPDH-R: CGACATGTGAGATCCACGAC
